# Bactericidal and antibiofilm activity of lactic acid bacteria-derived cell free extracts against dairy-associated spoilage and pathogenic bacteria

**DOI:** 10.3389/fmicb.2026.1783760

**Published:** 2026-03-02

**Authors:** Andressa Falqueto, Rafaela da Silva Rodrigues, Luana Virgínia Souza, Antônio Fernandes de Carvalho, Cinzia Caggia, Luiz Augusto Nero, Solimar Gonçalves Machado, Cinzia Lucia Randazzo

**Affiliations:** 1 InovaLeite - Laboratório de Pesquisa em Leites e Derivados, Departamento de Tecnologia de Alimentos, Universidade Federal de Viçosa, Viçosa, MG, Brazil; 2 Di3A - Dipartimento di Agricoltura, Alimentazione e Ambiente, Università degli Studi di Catania, Catania, Italy; 3 InsPOA - Laboratório de Inspeção de Produtos de Origem Animal, Departamento de Medicina Veterinária, Universidade Federal de Viçosa, Viçosa, MG, Brazil

**Keywords:** bacterial membrane disruption, biopreservative, food safety, food spoilage, growth inhibition, organic acid profile

## Abstract

This study aimed to evaluate the antimicrobial and antibiofilm potential of 15 LAB strains using five types of LAB-derived preparations: cell-free supernatants (CFS), sonicated-inactivated cells (IC), their combination (ICS), and their neutralized variants (CFS N, ICS N) to identify the most effective strain-extract combinations for potential application as natural biocontrol agents in dairy systems. Antimicrobial activity was assessed through agar diffusion assays, growth and biofilm inhibition, and determination of minimum inhibitory (MIC) and bactericidal (MBC) concentrations. The extracts were further characterized by pH and organic acid profiles using high-performance liquid chromatography (HPLC), and their mechanisms of action were investigated through cellular leakage assays, time–kill kinetics, and scanning electron microscopy (SEM). Several LAB-derived extracts exhibited strong antagonistic and antibiofilm activity against both spoilage and pathogenic bacteria. Non-neutralized CFS and ICS showed pronounced bactericidal activity, confirming the central role of organic acids in microbial inhibition. Z-score ranking and leakage assays identified *Lactiplantibacillus plantarum* Q4C3, *Lactococcus lactis* subsp. *lactis* biovar diacetylactis SBR4, *Weissella cibaria* W21, and *Weissella viridescens* W23 as the most effective strains. Time–kill assays demonstrated rapid microbial reductions (>3 log CFU/mL) within 4 h by CFS, whereas ICS required longer exposure. SEM analysis revealed severe membrane disruption in CFS-treated cells and the presence of LAB-derived debris surrounding ICS-exposed cells. These findings demonstrate that acidic LAB-derived extracts, particularly CFS, efficiently disrupt microbial cells and support their use as safe and effective natural biocontrol agents for improving the microbial safety and quality of dairy products.

## 1 Introduction

Microbiological contamination remains one of the major challenges in cheese production, where both pathogenic and spoilage microorganisms can compromise quality, reduce shelf life, and pose health risks to consumers (Fusco et al., 2020). Pathogens, such as *Salmonella* spp., *Listeria monocytogenes*, *Staphylococcus aureus*, and pathogenic *Escherichia coli* strains continue to be frequently reported in dairy products (Mantovam et al., 2025), with contamination often linked to failures during production, handling, or storage including inadequate hygiene during milking and herd management, insufficient cleaning and sanitation of equipment that promotes biofilm formation, improper temperature control and breaks in the cold chain, post-pasteurization contamination, and poor environmental management (Jayarao and Henning, 2001; Oliver et al., 2005; Kousta et al., 2010; Martin et al., 2023). Spoilage organisms, particulary *Pseudomonas* spp. and coliforms, also contribute to product deterioration through pigment formation, enzymatic degradation, and gas production, leading to undesirable sensory and structural changes (Martin et al., 2016; Kumar et al., 2019).

These challenges underscore the need for effective and natural strategies to control both pathogenic and spoilage organisms in dairy matrices. Lactic acid bacteria (LAB) play a central role in food fermentation and are well known for producing antimicrobial metabolites, including organic acids and bacteriocins, that inhibit undesirable microorganisms (Snyder et al., 2024). Recently, interest has expanded toward the use of LAB-derived extracts, which contain bioactive compounds obtained from inactivated cells and culture supernatants. Unlike probiotics or bio-preservative cultures that rely on the presence of viable microorganisms, LAB-derived extracts offer antimicrobial functionality through metabolites alone, making them attractive for clean-label food applications (Raman et al., 2022; Banicod et al., 2025; Choi et al., 2024).

In this context, preparations obtained from LAB are frequently discussed in the literature within the framework of postbiotics. However, according to the International Scientific Association for Probiotics and Prebiotics (ISAPP), postbiotics require demonstrated health benefit to the host (Salminen et al., 2021). When no host-related effects are addressed to the LAB evaluated strains, the preparations are not classified as postbiotics and are instead framed as LAB-derived antimicrobial extracts intended for food biocontrol applications. These preparations include cell-free supernatants, culture media enriched with extracellular metabolites, and intracellular components released after cell inactivation, representing a diverse set of bioactive microbial products (Karabacak Aydin et al., 2026).

Although combined physical, chemical, and biological treatments often enhance microbial inactivation (Ashrafudoulla et al., 2024), the synergistic action of multiple metabolites within a single LAB-derived extract may also potentiate antimicrobial effects (Soltani et al., 2022; Teneva and Denev, 2023). LAB-derived compounds have demonstrated promising activity against biofilm formation and microbial contamination in food systems, aligning with the growing demand for natural and minimally processed products (Yildirim et al., 2018; Moradi et al., 2020).

Comparative evaluations of multiple LAB strains and their derived extracts, particularly in the context of foodborne pathogens and dairy-related spoilers, are still limited. Furthermore, the relative contribution of organic acids, cell-associated components, and other metabolites to the antimicrobial activity of different extract preparations is not yet fully understood. Evaluating extracts containing only supernatants, only inactivated cells, or combinations of both, either native or neutralized, can help clarify the mechanisms underlying microbial inhibition and support their rational application in dairy systems.

Accordingly, this study aimed to comparatively screen 15 LAB strains for antimicrobial and antibiofilm activity against dairy-associated spoilage and pathogenic microorganisms. Five types of LAB-derived preparations (cell-free supernatants, inactivated cells, their combination, and their neutralized variants) were systematically evaluated to determine the contribution of organic acids and cell-associated components to microbial inhibition. This approach enabled the identification and selection of the most effective strain–extract combinations with potential application as natural biocontrol agents in dairy systems. Although antimicrobial activity of LAB-derived cell-free supernatants has been widely reported, most studies focus on a limited number of strains and extract types. The novelty of the present work lies in the systematic, strain-level comparison of 15 LAB strains using five distinct extract preparations, including neutralized variants, combined with an integrated functional and mechanistic evaluation. By linking antimicrobial and antibiofilm performance to organic acid profiles, membrane damage, and killing kinetics, this study provides a rational framework for selecting LAB-derived extracts with enhanced efficacy and industrial relevance, rather than reporting isolated antimicrobial effects.

## 2 Materials and methods

### 2.1 Microorganisms and growth conditions

Fifteen strains of LAB and seven strains of pathogenic and spoilage microorganisms (hereafter referred to as contaminants) were used (Table 1). The contaminants strains were maintained at −80 °C (±2 °C) in Brain Heart Infusion (BHI) broth (Kasvi, Curitiba, Brasil) while the LAB were maintained at the same temperature in Man, Rogosa and Sharpe (MRS) broth (Kasvi, Curitiba, Brasil). The culture medium was supplemented with 20% (v/v) glycerol.

**Table 1:** Lactic acid bacteria (LAB), pathogens and spoilage strains used in this work and their isolation sources.

Microorganisms	Source	Growth conditions	Abbreviations	Assays performed	References
LAB strains					
*Lactiplantibacillus plantarum* Q4C3	Artisanal cheese/Ilha do Marajó – Pará, Brazil	Cultured in MRS^a^ broth or agar at 30 °C (±2 °C) for 24–48 h	*Lpb. plantarum* Q4C3	Antagonistic/antibiofilm/MIC/MBC/cell-leakage/Time–kill/SEM	Souza et al. (2023)
*Lactococcus lactis* subsp. *lactis* biovar diacetylactis SBR4	Silage		*Lc.* subsp. *lactis* biovar diacetylactis SBR4	Antagonistic/antibiofilm/MIC/MBC/cell-leakage/Time–kill/SEM	Fusieger et al. (2020b)
*Lactococcus lactis* subsp. *lactis* Lc08	Raw Milk		*Lc. lactis* subsp. *lactis* Lc08	Antagonistic/antibiofilm/MIC/MBC/cell-leakage/Time–kill	Perin et al. (2013)
*Weissella viridescens* W23	Pasture/Campos das vertentes – Minas Gerais, Brazil		*W. viridescens* W23	Antagonistic/antibiofilm/MIC/MBC/cell-leakage/Time–kill/SEM	Teixeira et al. (2021)
*Weissella confusa* W8	Raw milk /Ilha do Marajó – Pará, Brazil		*W. confusa* W8	Antagonistic and antibiofilm activity	
*Weissella paramesenteroides* W11	Artisanal cheese/Sul do Pará - Pará, Brazil		*W. paramesenteroides* W11	Antagonistic and antibiofilm activity	
*Weissella paramesenteroides* W10	Artisanal cheese/Ilha do Marajó - Pará, Brazil		*W. paramesenteroides* W10	Antagonistic and antibiofilm activity	
*Weissela cibaria* W42	Soil/Campos das vertentes – Minas Gerais, Brazil		*W. cibaria* W42	Antagonistic and antibiofilm activity	
*Weissela cibaria* W32			*W. cibaria* W32	Antagonistic and antibiofilm activity	
*Weissela cibaria* W49	Silage/Campos das vertentes – Minas Gerais, Brazil		*W. cibaria* W49	Antagonistic and antibiofilm activity	
*Weissela cibaria* W21	Pasture/Campos das vertentes - Minas Gerais, Brazil		*W. cibaria* W21	Antagonistic/antibiofilm/MIC/MBC/cell-leakage/Time–kill/SEM	
*Weissela cibaria* W22			*W. cibaria* W22	Antagonistic/antibiofilm/MIC/MBC/cell-leakage	
*Weissella cibaria* W25			*W. cibaria* W25	Antagonistic and antibiofilm activity	
*Lactococcus lactis* subsp*. lactis* biovar diacetylactis ATCC 13675	Culture collection		*Lc. lactis* subsp*. lactis* biovar diacetylactis ATCC 13675	Antagonistic/antibiofilm/MICC/MBC/cell-leakage	ATCC^*^
*Lactococcus lactis* subsp. *lactis* ATCC 19435	Raw milk, culture collection		*Lc. lactis* subsp. *Lactis* ATCC 19435	Antagonistic/antibiofilm/MIC/MBC/cell-leakage	ATCC^*^
Pathogen strains					
*Salmonella Typhi*murium ATCC 14028	Pooled heart and liver tissue of four-week-old chickens	Cultured in BHI^b^ broth or agar at 37 °C (±2 °C) for 24 h	*S. typhimurium* ATCC 14028	Antagonistic/antibiofilm/MIC/MBC/cell-leakage	ATCC^*^
*Listeria monocytogenes* ATCC 19117	Sheep		*L. monocytogenes* ATCC 19117	Antagonistic/antibiofilm/MIC/MBC/cell-leakage/Time–kill/SEM	ATCC^*^
*Staphylococcus aureus* ATCC 19095	Patient’s leg abscess		*S. aureus* ATCC 19095	Antagonistic/antibiofilm/MIC/MBC/cell-leakage	ATCC^*^
*Escherichia coli* O157: H7	Culture collection		*E. coli* O157: H7	Antagonistic/antibiofilm/MIC/MBC/cell-leakage	InovaLeite^**^
Spoilage strains					
*Pseudomonas paracarnis* A006	Fresh Cheese/MG	Cultured in BHI^b^ broth or agar at 25 °C (±2 °C) for 24 h	*P. paracarnis* A006	Antagonistic/antibiofilm/MIC/MBC/cell-leakage	Rodrigues et al. (2021)
*Pseudomonas fluorescens* 07A	Raw Milk		*P.fluorescens* 07A	Antagonistic/antibiofilm/MIC/MBC/cell-leakage/Time–kill/SEM	Pinto et al. (2004)
*Escherichia coli* 1791	Raw Milk	Cultured in BHI^b^ broth or agar at 37 °C (±2 °C) for 24 h	*E. coli* 1791	Antagonistic/antibiofilm/MIC/MBC/cell-leakage	INSPOA^***^

^a^MRS, Man, Rogosa and Sharpe; ^b^BHI, Brain Heart Infusion; ^*^ATCC: American Type Culture Collection; ^**^Milk and Dairy Products Research Laboratory- InovaLeite; ^***^Laboratório de Inspeção de Produtos de Origem Animal – INSPOA. MIC, Minimum inhibitory concentration; MBC, Minimum bactericidal concentration. SEM - Scanning Electron Microscopy.

Before each experiment, the stock cultures stored at −80 °C (±2 °C) were refreshed by inoculating 1% (v/v) into fresh medium (Table 1). Incubation was performed under the optimal growth conditions for each microorganism under static conditions (Table 1). After growth, cell suspensions were diluted in 0.85% (w/v) saline solution to an optical density (OD) at 600 nm of 0.200 to 0.280 corresponding to approximately 1.5 × 10^8^ CFU/mL. OD was measured using a UV-5100 UV–Vis spectrophotometer (Global Trade Technology, China). The bacterial population in the standardized cell suspensions was confirmed by plate counting on solid media using the microdrop technique (Morton, 2001), followed by incubation under the conditions provided for each microorganism (Table 1).

### 2.2 Preparation of LAB-derived extracts

LAB-derived extracts evaluated in this study comprised cell-free supernatants (CFS), inactivated cells (IC), their combination (ICS), and their neutralized variants (CFS N, ICS N) prepared as described below.

Cell-free supernatants (CFS) were obtained from the 15 standardized LAB suspensions (1.5 × 10^8^ CFU/mL) by centrifugation at 10,000 g for 10 min at 4 °C. The supernatants were filtered through 0.22 μm pore-size membranes to obtain the CFS which was stored frozen (−20 °C) until use (İncili et al., 2021). For inactive cells plus cell-free supernatants (ICS), standardized LAB suspensions were sonicated for 10 min in an ice bath using a probe sonicator (Ultrasonic Probe System QR850, Ecosonics, Brazil) operating at 40 kHz and 100% amplitude. Similarly, to obtain the inactive cells (IC), the standardized LAB suspensions were centrifuged at 10,000 g at 4 °C for 15 min. The recovered pellets were washed three times, resuspended in sterile distilled water and sonicated under the same conditions used for ICS (Sharafi et al., 2023). The neutralized cell free supernatant (CFS N) and the neutralized inactive cells plus cell-free supernatant (ICS N) were prepared in the same way, with 1 M NaOH added to adjust the pH to approximately 7.0. Cell death was confirmed for all extracts by plating them under optimal conditions (Table 1), as well as for the negative growth control in all subsequent analyses.

Once obtained, the extracts had their pH measured. The measurements were conducted according to Fusieger et al. (2020a), using a calibrated digital pH meter (Hanna Instruments, São Paulo, SP, Brazil) and the extracts were subsequently stored at −20 °C.

### 2.3 Antibacterial activity

#### 2.3.1 Agar diffusion method

The LAB antimicrobial activity was screened using a modified colony overlay method as described by Jeong et al. (2024). Briefly, a loopful of the revived LAB cultures was streaked onto MRS agar plates which was left half-open to dry for 1 h under aseptic conditions. The plates were overlaid with 10 mL of previously homogenized BHI agar (cooled to approximately 45 °C) containing each pathogenic or spoilage strain at a final population of 10^7^ CFU/mL. After incubation for 24–48 h under the optimal conditions of each bacteria (Table 1), the presence of an inhibition zone (a clear halo ≥10 mm in diameter from the streak edge), was considered indicative of positive antagonistic activity.

#### 2.3.2 Antagonistic and antibiofilm activity

To the antagonistic activity, the extracts were tested against pathogens and spoilage organisms using a microplate assay. Aliquots of 90 μL of BHI broth (1×) were mixed with an equal volume of each LAB-derived extract in 96-well microtiter plates. Then, each well was inoculated with 20 μL of contaminant cell suspensions standardized to 10^6^ CFU/mL. The plates were incubated at 37 or 25 °C, depending on the inoculated microorganism (Table 1), for 24 h in a microplate spectrophotometer (Multiskan™ GO, Thermo Fisher, Scientific Inc., Finland). The OD was measured at 600 nm every 60 min for 24 h (Arena et al., 2016).

Using the same incubation conditions employed for the antagonistic activity, the antibiofilm activity of LAB-derived extracts was evaluated as described by Kim et al. (2022), with minor modifications. Briefly, contaminants were co-incubated with the LAB-derived extracts in 96-well microtiter plates under the conditions described above, allowing biofilm formation in the presence of the extracts for 24 h. After incubation, the culture was carefully removed, and the wells were gently washed three times with phosphate-buffered saline (PBS; pH 7.4; Sigma-Aldrich) to remove non-adhered cells. The adhered biofilm was stained with 200 μL of 0.1% (w/v) crystal violet (Sigma-Aldrich) at 25 °C (±2 °C) for 20 min. Excess stain was removed by washing the wells three times with PBS, followed by drying at 37 °C for 20 min. Subsequently, 200 μL of 95% (v/v) ethanol (Sigma-Aldrich) was added to solubilize the bound dye for 15 min at room temperature. Biofilm biomass was quantified by measuring absorbance at 590 nm (OD590) using a microplate spectrophotometer (Multiskan™ GO, Thermo Fisher Scientific Inc., Finland).

Positive controls consisted of wells containing the contaminants cell suspensions (10^6^ CFU/mL) and BHI broth without LAB-derived extracts, whereas negative controls contained BHI broth supplemented with the corresponding LAB-derived extract.

Absorbance values were baseline-corrected by subtracting the negative controls, and biofilm inhibition (%) was calculated relative to the positive control according to Equation 1. The OD590 values of the positive controls (ODc) were compared with those of the treated cultures (ODt), and biofilm inhibition (%) was calculated according to Equation 1.

$$\text{Inhibi}\text{tion}\;(\%)=100-(OD_{t}\;x\;\frac{100}{OD_{c}})$$ (1)

The results obtained from the antagonistic activity and antibiofilm assays were normalized using Z-scores, calculated according to Equation 2 (Du et al., 2022), where X represents the observed value, *μ* the dataset mean, and *σ* the standard deviation. The Z-score indicates how many standard deviations a value deviates from the mean, facilitating data interpretation. Z-score values were considered to rank the LAB-derived extracts, prioritizing those that showed Z-score < 1 (less than one standard deviation above or below the population mean).

$$Z-\text{score}=\frac{\left(X-\mu \right)}{\;\sigma }$$ (2)

All experiments were carried out in triplicate with two independent biological replicates. The LAB-derived extracts from the eighth strains showing the strongest antimicrobial and antibiofilm activity were selected for further analysis.

### 2.4 Minimum inhibitory concentration (MIC) and minimum bactericidal concentration (MBC)

The MIC and MBC of LAB-derived extracts were determined based on the microplate dilution technique detailed by Sadekuzzaman et al. (2018) with modifications. The LAB-derived extracts were diluted in BHI to obtain concentrations: 100 (no dilution), 75, 50, 25 and 12.5% (v/v) and distributed in the 96-well microplate (180 μL/well). Aliquots of 20 μL of contaminant cell suspension, adjusted to 10^5^ CFU/mL were added to each well. Plates were incubated at the optimal growth temperature for each evaluated strain (Table 1), and OD measurements at 600 nm were recorded after 24 h using a microplate spectrophotometer (MultiscanGo, ThermoFisher, Finland). Controls included fresh BHI broth (blank), BHI broth inoculated with contaminants without LAB-derived extracts (positive control), and BHI broth with LAB-derived extracts without contaminants (negative control). All controls were incubated under the same conditions as the test samples. The MIC was defined as the lowest extract concentration that completely inhibited bacterial growth (ΔD ≈ 0). To determine MBC, the content of each well was plated on BHI agar using microdrop technique (Morton, 2001). The Petri dishes were incubated as described in Table 1. The MBC was defined as the lowest LAB-derived extract concentration that prevented colony formation. The experiments were carried out in triplicate with two independent biological replicates. For the subsequent characterization of organic acid profiles and the further assays, extracts that demonstrated bactericidal activity were selected.

### 2.5 Characterization of the organic acid profile

Organic acid analysis was performed using a high-performance liquid chromatography system (HPLC; Shimadzu, São Paulo, Brazil) equipped with a refractive index detector (RID-20A), following the methodology described by Zhang et al. (2024), with minor modifications. Initially, the selected LAB-derived extracts were centrifuged at 10,000×*g* for 15 min at 4 °C and subsequently filtered through a 0.22 μm pore-size membrane filter. A 10 μL aliquot of each sample was injected into an Aminex® HPX-87H column (300 mm × 7.8 mm), coupled with a guard column (Bio-Rad Laboratories, Rio de Janeiro, Brazil). The mobile phase consisted of 0.005 mol/L sulfuric acid (H₂SO_4_) in ultrapure water, delivered at a constant flow rate of 0.7 mL/min. The column was maintained at 45 °C, and the total run time for each analysis was 30 min. Chromatographic data were acquired and processed using LabSolutions software (Shimadzu Corporation, 2013). Standards of lactic, citric, succinic, formic, acetic, propionic, isobutyric, butyric, isovaleric, and valeric acids were used to identify the chromatogram peaks, compare the retention times, and calculate their concentration in the samples. One sample of each extract was analyzed in duplicate.

### 2.6 Cell leakage assay

Cell leakage assessment was performed following Sulistyani et al. (2023) and Wang and Zeng (2022), with adaptations. Ten milliliters of bacterial suspensions (10^8^ CFU/mL) of *P. paracarnis* A006, *P. fluorescens* 07A, *E. coli* 1791, *E. coli* O157: H7, *L. monocytogenes* ATCC 19117, *S. aureus* ATCC 19095, and *S. typhimurium* ATCC 14028 were centrifuged for 20 min at 4,500 rpm. The pellet was collected and washed three times with PBS (pH 7.4), then resuspended in LAB-derived extracts that demonstrated bactericidal activity against at least one contaminant at 1 × MBC. The mixture was incubated as described in Table 1 for 24 h. Subsequently, the samples were centrifuged for 20 min at 4,500 rpm, and the absorbance of the supernatant was measured at 260 and 280 nm to detect nucleic acids (DNA and RNA) using a Shimadzu UV-1800 UV–Vis Spectrophotometer (Shimadzu, Japan). DNA and RNA concentrations were calculated according to Equations 3 and 4, respectively. The LAB-derived extracts from four strains exhibiting the highest potential for cellular damage, leading to nucleic acid leakage, were selected for the time-kill assay and scanning electron microscopy (SEM) imaging. The experiments were carried out in triplicate with two independent biological replicates.

DNAconcentration(μg/mL)=A260×50 (3)

RNAconcentration(μg/mL)=A260×40 (4)

### 2.7 Time-kill assay

The time-kill assay was conducted following the method described for Sulistyani et al. (2023). The contaminants *P. fluorescens 07A* and *L. monocytogenes* ATCC 19117 were selected as target microorganisms. BHI broth containing both contaminants at 10^5^ CFU/mL was mixed with the LAB-derived extract at a concentration of 1×MBC, and then incubated as mentioned in Table 1 for 0, 2, 4 and 6 h. An aliquot of 20 μL of each sample was collected and inoculated with a pour plate method using BHI agar and then incubated at 37 °C for 18–24 h. The colonies were counted and then time-kill curves were constructed by plotting log_10_ CFU/mL against time (h). The experiments were carried out in triplicate with two independent biological replicates.

### 2.8 SEM analysis

The morphological changes in planktonic cells of *P. fluorescens* 07A and *L. monocytogenes* ATCC 19117 treated with CFS and ICS were evaluated by SEM, as previously described by Jiang et al. (2022). Initially, 1 mL of *L. monocytogenes* ATCC 19117 and *P. fluorescens* 07A cell suspension (10^7^ CFU/mL) was centrifuged at 6,000 rpm for 5 min at 4 °C, followed by three washing steps with PBS. The recovered cells were then treated with the LAB-derived extracts at 1 × MBC and incubated following the conditions described in Table 1 for 4 h. After treatment, the cell suspension was centrifuged again, washed three times with PBS and fixed on brass stubs with 2.5% glutaraldehyde at 4 °C for 8 h. The samples were dried in a desiccator at room temperature for 7 days (no dehydration step was performed), sputter-coated with gold (Quorum—Q150TES Plus, East Sussex, England), and examined using a scanning electron microscope (JSM-IT700HR, JEOL, Tokyo, Japan). Untreated cells were used as controls.

### 2.9 Statistical analysis

Statistical analyses and data visualization were performed using R software version 4.3.3 and RStudio 2023.03.0 + 386 (RStudio Team, 2023; R Core Team, 2024). The Shapiro–Wilk test and Bartlett’s test were used to assess the normality of residuals and the homogeneity of variances, respectively. For the antagonism assays, an analysis of variance (ANOVA) was performed for each of the seven target microorganisms within each LAB studied, considering the six evaluated treatments. Tukey’s test was applied as a *post hoc* procedure. For the time-kill assay, statistical analysis was conducted using the Kruskal–Wallis test followed by Conover’s test, whereas cell leakage data were analyzed using ANOVA and Tukey’s test. Statistical significance was set at *p* < 0.05.

## 3 Results

### 3.1 Antagonistic effects and prevention of biofilm formation

**Figure 1:**
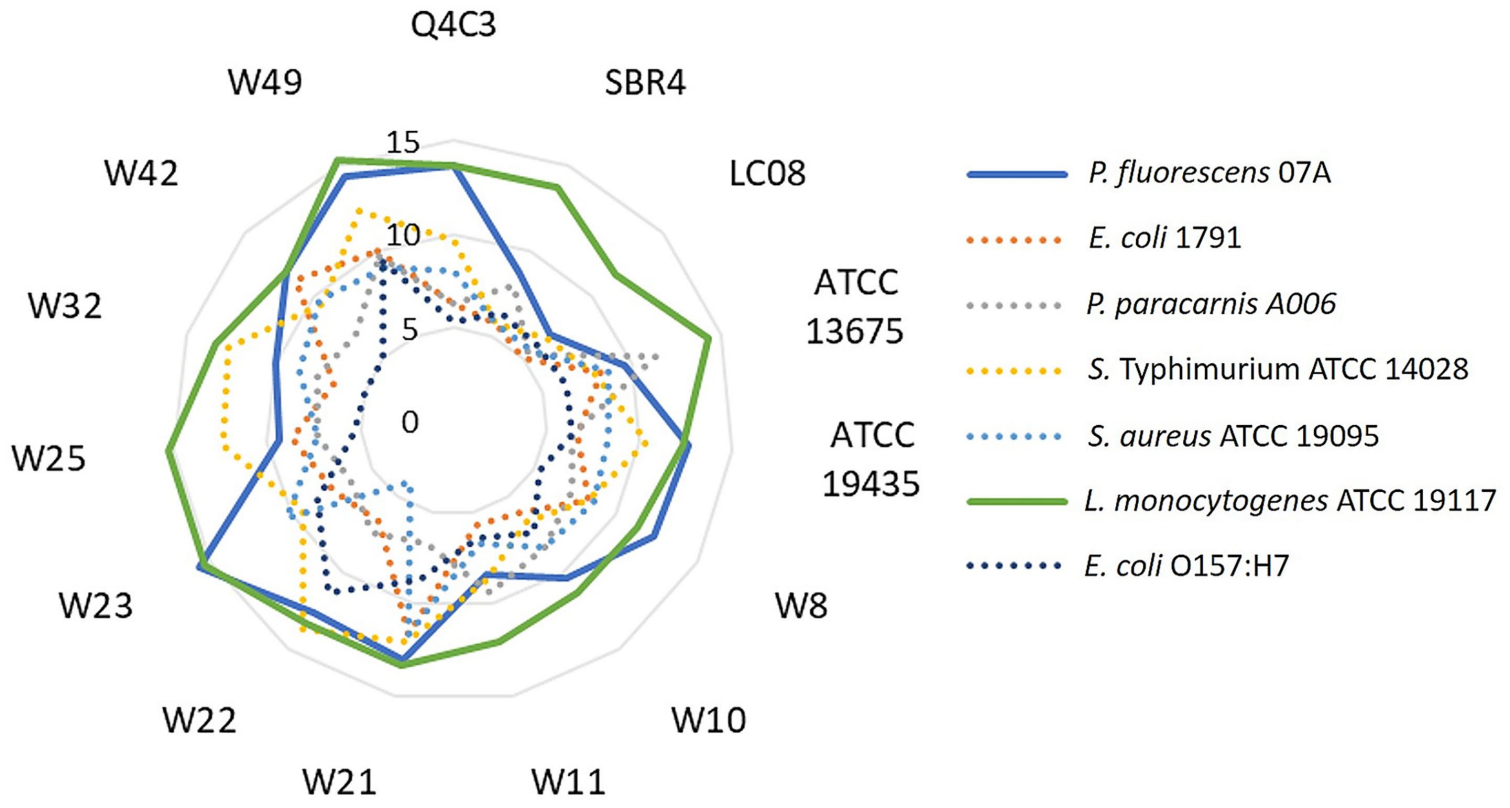
Radar chart illustrating the inhibition halo diameters (mm) generated by *Lpb. plantarum* Q4C3, *Lc. lactis* subsp. *lactis* biovar diacetylactis SBR4, *Lc. lactis* subsp. *lactis* Lc08, *W. viridescens* W23, *W. confusa* W8, *W. paramesenteroides* W11, *W. paramesenteroides* W10, *W. cibaria* W42, *W. cibaria* W32, *W. cibaria* W49, *W. cibaria* W21, *W. cibaria* W22, *W. cibaria* W25, *Lc. lactis* subsp. *lactis* biovar diacetylactis ATCC 13675, *Lc. lactis* subsp. *lactis* ATCC 19435 against the contaminants: *Salmonella* Typhimurium ATCC 14028, *L. monocytogenes* ATCC 19117, *S. aureus* ATCC 19095, *E. coli* O157: H7, *P. paracarnis* A006*, P. fluorescens* 07A, *E. coli* 1791, after 24 h of incubation in the agar diffusion assay. Each value represents the mean of triplicate.

Antibacterial activity of fifteen LAB strains against seven food-related spoilage and pathogenic microorganisms was initially assessed using agar diffusion assays. *L. monocytogenes* ATCC 19117 and *P. fluorescens* showed the largest inhibition zones (Figure 1), with inhibition patterns varying among strains.

Following the initial screening by agar diffusion assays, the antimicrobial potential of LAB-derived extracts was further assessed through growth inhibition and antibiofilm assays. Table 2 presents the pH values of the LAB-derived extracts. The pH ranged from 3.84 to 4.46 for CFS, 6.96 to 7.18 for CFS N, 3.87 to 4.61 for ICS, 6.65 to 7.15 for ICS N, and 6.02 to 6.37 for IC.

**Table 2:** pH values of lactic acid bacteria-derived extracts produced under different experimental conditions.

LAB	CFS	CFS N	ICS	ICS N	IC
*Lactiplantibacillus plantarum* Q4C3	4.46 ± 0.11	7.04 ± 0.2	4.52 ± 0.01	6.96 ± 0.016	6.37 ± 0.11
*Lactococcus lactis* subsp. *lactis* biovar diacetylactis SBR4	4.18 ± 0.06	7.18 ± 0.01	4.35 ± 0.02	6.89 ± 0.07	6.21 ± 0.15
*Lactococcus lactis* subsp. *lactis* Lc08	4.11 ± 0.30	6,98 ± 0.04	4.61 ± 0.04	6.94 ± 0.07	6.19 ± 0.04
*Weissella viridescens* W23	4.36 ± 0.41	7.06 ± 0.03	4.32 ± 0.04	6.89 ± 0.13	6.22 ± 0.06
*Weissella confusa* W8	4.03 ± 0.13	7.02 ± 0.01	4.23 ± 0.01	6.65 ± 0.24	6.17 ± 0.07
*Weissella paramesenteroides* W11	3.84 ± 0.06	7.12 ± 0.01	3.87 ± 0.04	6.85 ± 0.08	6.02 ± 0.08
*Weissella paramesenteroides* W10	4.08 ± 0.01	7.06 ± 0.01	4.19 ± 0.01	7.02 ± 0.04	6.21 ± 0.14
*Weissella cibaria* W42	4.33 ± 0.17	6.96 ± 0.04	4.32 ± 0.04	7.02 ± 0.01	6.26 ± 0.16
*Weissella cibaria* W32	4.13 ± 0.10	7.17 ± 0.04	4.10 ± 0.02	6.92 ± 0.11	6.18 ± 0.08
*Weissella cibaria* W49	4.33 ± 0.03	7.18 ± 0.01	4.22 ± 0.02	7.15 ± 0.15	6.16 ± 0.04
*Weissella cibaria* W21	4.45 ± 0.10	7.18 ± 0.04	4.27 ± 0.06	7.06 ± 0.11	6.30 ± 0.10
*Weissella cibaria* W22	4.40 ± 0.10	7.13 ± 0.05	4.23 ± 0.05	6.94 ± 0.08	6.24 ± 0.14
*Weissella cibaria* W25	4.39 ± 0.06	7.15 ± 0.02	4.25 ± 0.08	7.12 ± 0.06	6.28 ± 0.14
*Lactococcus lactis* subsp*. lactis* biovar diacetylactis ATCC 13675	4.43 ± 0.16	6.98 ± 0.08	4.53 ± 0.03	7.15 ± 0.06	6.23 ± 0.15
*Lactococcus lactis* subsp. *lactis* ATCC 19435	4.37 ± 0.04	6.97 ± 0.07	4.51 ± 0.08	7.01 ± 0.08	6.21 ± 0.12

CFS, Cell-free supernatants; ICS, inactive cells plus cell-free supernatants; CFS N, neutralized cell free supernatant; ICS N, neutralized inactive cells plus cell-free supernatant; IC, inactive cells.

**Figure 2:**
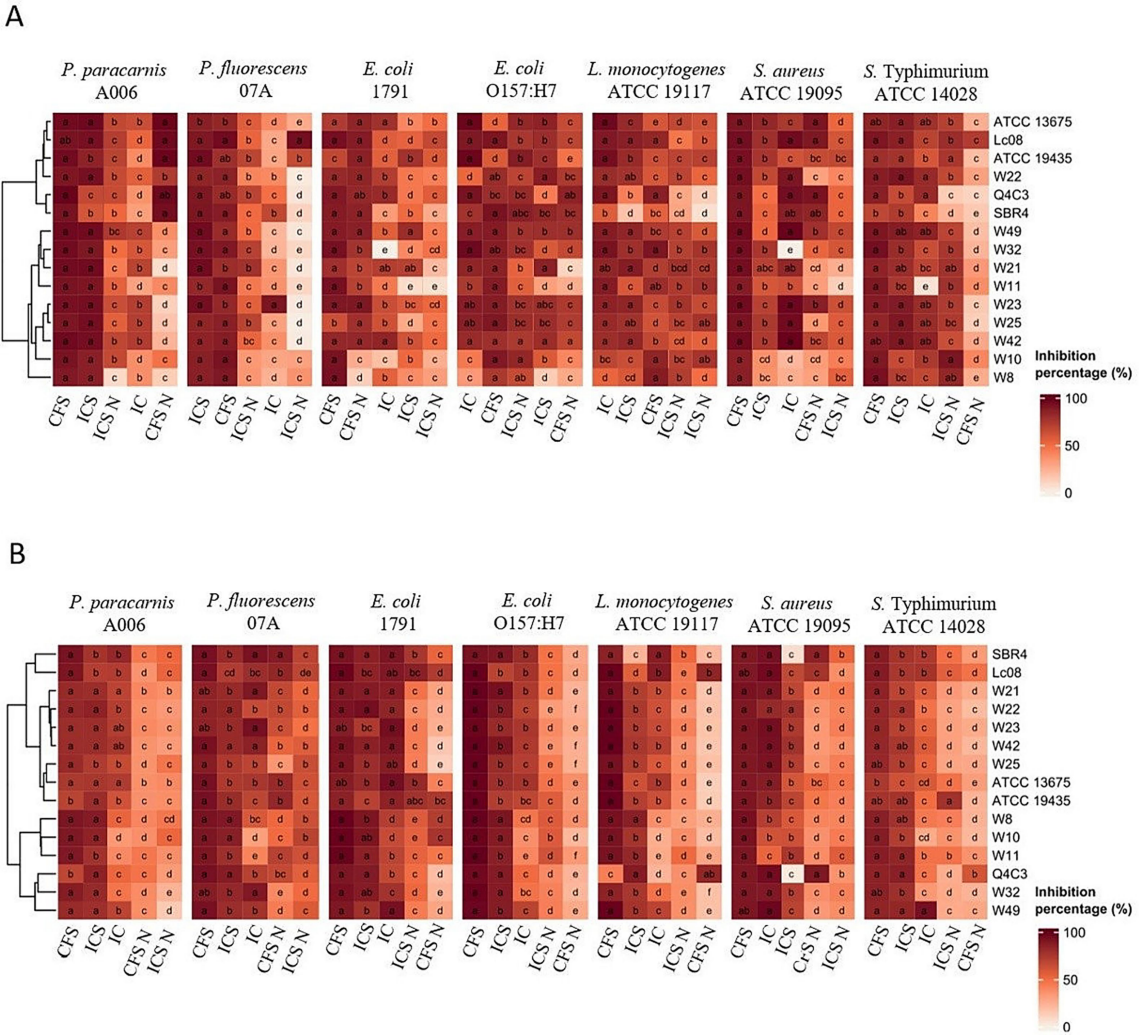
Heatmap of growth inhibition (%) (**A**: OD600) and biofilm inhibition (%) (**B**: OD590) of *P. paracarnis* A006, *P. fluorescens* 07A, *E. coli* 1791, *E. coli* O157: H7, *L. monocytogenes* ATCC 19117, *S. aureus* ATCC 19095, and *S.* typhimurium ATCC 14028 in response to six treatments: CFS, Cell-free supernatants; ICS, inactive cells plus cell-free supernatants; CFS N, neutralized cell free supernatant; ICS N, neutralized inactive cells plus cell-free supernatant, and IC, inactive cells, prepared from *Lpb. plantarum* Q4C3, *Lc. lactis* subsp. *lactis* biovar diacetylactis SBR4, *Lc. lactis* subsp. *lactis* Lc08, *W. viridescens* W23, *W. confusa* W8, *W. paramesenteroides* W11, *W. paramesenteroides* W10, *W. cibaria* W42, *W. cibaria* W32, *W. cibaria* W49, *W. cibaria* W21, *W. cibaria* W22, *W. cibaria* W25*, Lc. lactis* subsp. *lactis* biovar diacetylactis ATCC 13675, *Lc. lactis* subsp. *lactis* ATCC 19435. The colors of the heatmaps agree with the percent inhibition. Letters indicate statistically significant differences between treatments (columns) in each lactic acid bacteria, based on Tukey’s test (*p* < 0.05). Tree densities represent hierarchical clustering applying the Euclidean distance matrix calculation and the Ward D2 method.

After 24 h of incubation, the LAB-derived extracts from *Lc. lactis Lc. lactis*subsp. *Lactis* biovar diacetylactis ATCC 13675, *Lc. lactis Lc. lactis*subsp. *Lactis* Lc08, *Lc. lactis Lc. lactis*subsp. *Lactis* ATCC 19435, *W. cibaria* W22, and *Lpb. plantarum* Q4C3 exhibited the highest inhibition of microbial growth (Figure 2A). For biofilm inhibition (Figure 2B), the most effective extracts were those derived from *Lc. lactis* subsp. *lactis* biovar diacetylactis SBR4, *Lc. lactis* subsp. *lactis* Lc08, *W. cibaria* W21, *W. cibaria* W22, and *W. viridescens* W23.

Z-score analysis (Supplementary material) identified *Lpb. plantarum* Q4C3, *Lc. lactis* subsp. *lactis* biovar diacetylactis ATCC 13675, *Lc. lactis* subsp. *lactis* ATCC 19435, *Lc. lactis* subsp. *lactis* Lc08, and *W. cibaria* W21 as the strains with the most consistent inhibitory performance across assays. This standardized comparison also indicated that CFS and ICS were the most effective extract types overall.

### 3.2 Minimum inhibitory concentration (MIC) and minimum bactericidal concentration (MBC)

**Figure 3:**
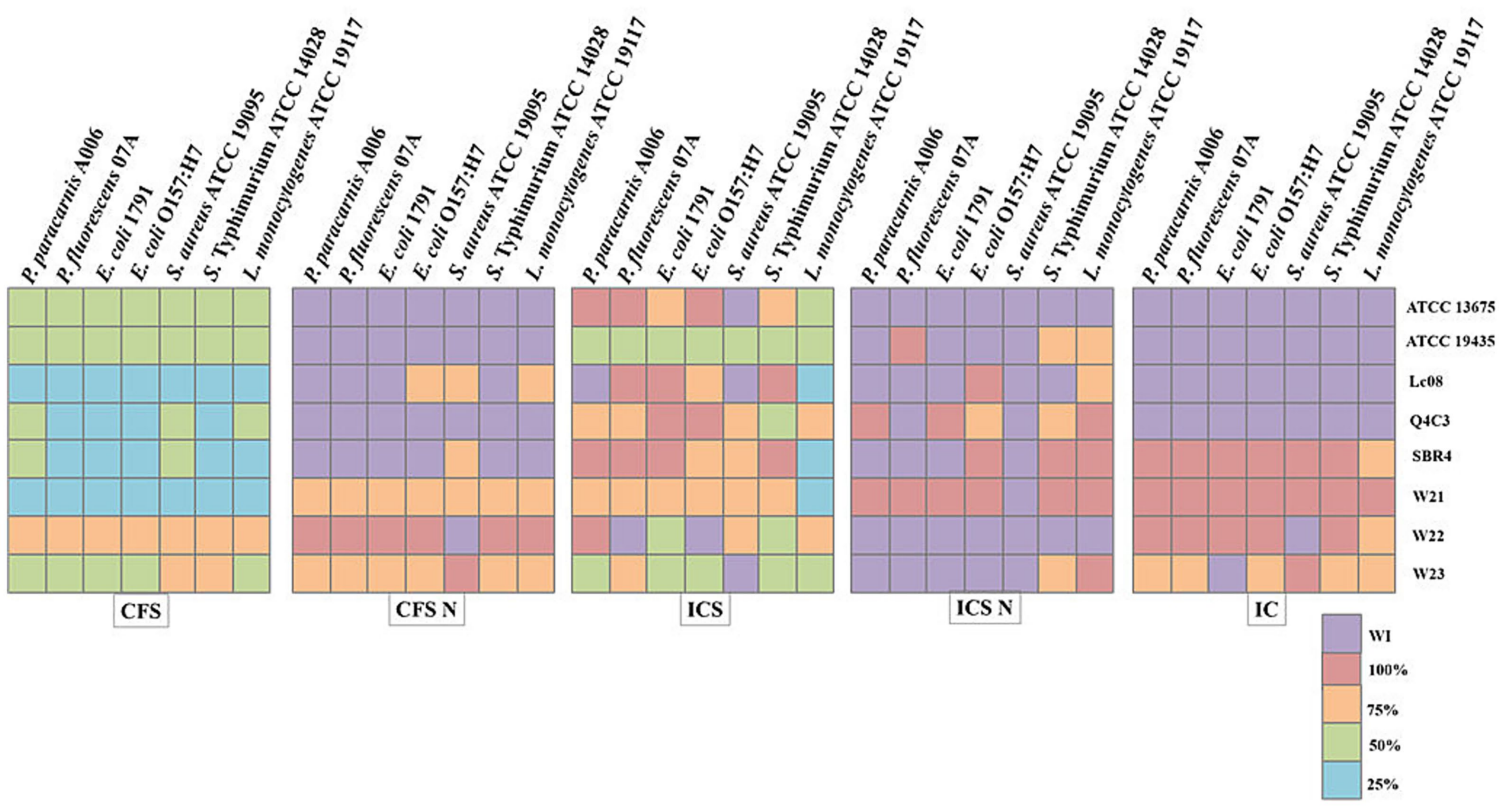
Minimum inhibitory concentration (MIC) values of LAB extracts in percentage determined against foodborne pathogens and spoilage microorganisms. CFS, Cell-free supernatants; ICS, inactive cells plus cell-free supernatants; CFS N, neutralized cell free supernatant; ICS N, neutralized inactive cells plus cell-free supernatant; IC, inactive cells and WI, without inhibition. *Lactococcus lactis* subsp. *lactis* biovar diacetylactis ATCC 13675, *Lactococcus lactis* subsp. *lactis* ATCC 19435, *Lactplantibacillus plantarum* Q4C3, *Lactococcus lactis* subsp. *lactis* biovar diacetylactis SBR4, *Lactococcus lactis* subsp. lactis Lc08, *Weissela cibaria* W21, *Weissela cibaria* W22, *Weissella viridescens* W23.

**Figure 4:**
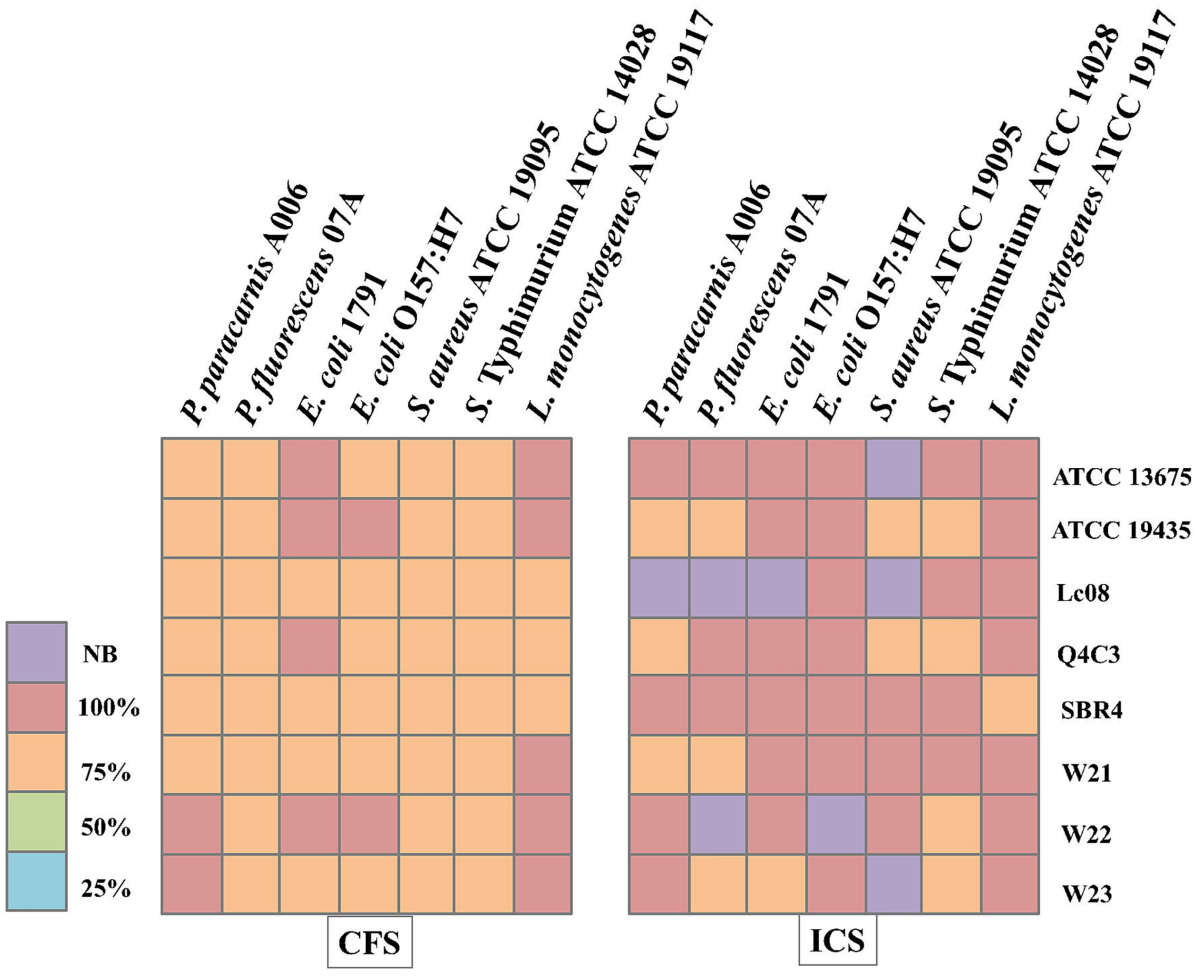
Minimum bactericidal concentration (MBC) values determined against foodborne pathogens and spoilage microorganisms. CFS, Cell-free supernatants; ICS, inactive cells plus cell-free supernatants; CFS N, neutralized cell free supernatant; ICS N, neutralized inactive cells plus cell-free supernatant; IC, inactive cells, and NB, Non-bactericida *Lactococcus lactis* subsp. *lactis* biovar diacetylactis ATCC 13675, *Lactococcus lactis* subsp. *lactis* ATCC 19435, *Lactplantibacillus plantarum* Q4C3, *Lactococcus lactis* subsp. *lactis* biovar diacetylactis SBR4, *Lactococcus lactis* subsp. *lactis* Lc08, *Weissela cibaria* W21, *Weissela cibaria* W22, *Weissella viridescens* W23.

Figure 3 shows the MIC values of the LAB-derived extracts against representative foodborne pathogens and spoilage microorganisms. CFS and ICS exhibited the lowest MIC values (25–100%), depending on the strain and target species. Lower MIC values (25–50%) were most frequently observed for extracts from *Lpb. plantarum* Q4C3, *Lc. lactis* subsp. *lactis* Lc08, and *W. cibaria* W21. In contrast, neutralized extracts (CFS N and ICS N) displayed reduced or no inhibitory activity (WI) for most target microorganisms. Only extracts from *W. cibaria* W21, *W. cibaria* W22, and *W. viridescens* W23 retained partial inhibitory capacity after neutralization. Among the evaluated extracts, only CFS and ICS displayed bactericidal activity (Figure 4). These treatments were therefore selected for subsequent cell leakage, time–kill, and SEM analyses.

### 3.3 Cell leakage

**Figure 5:**
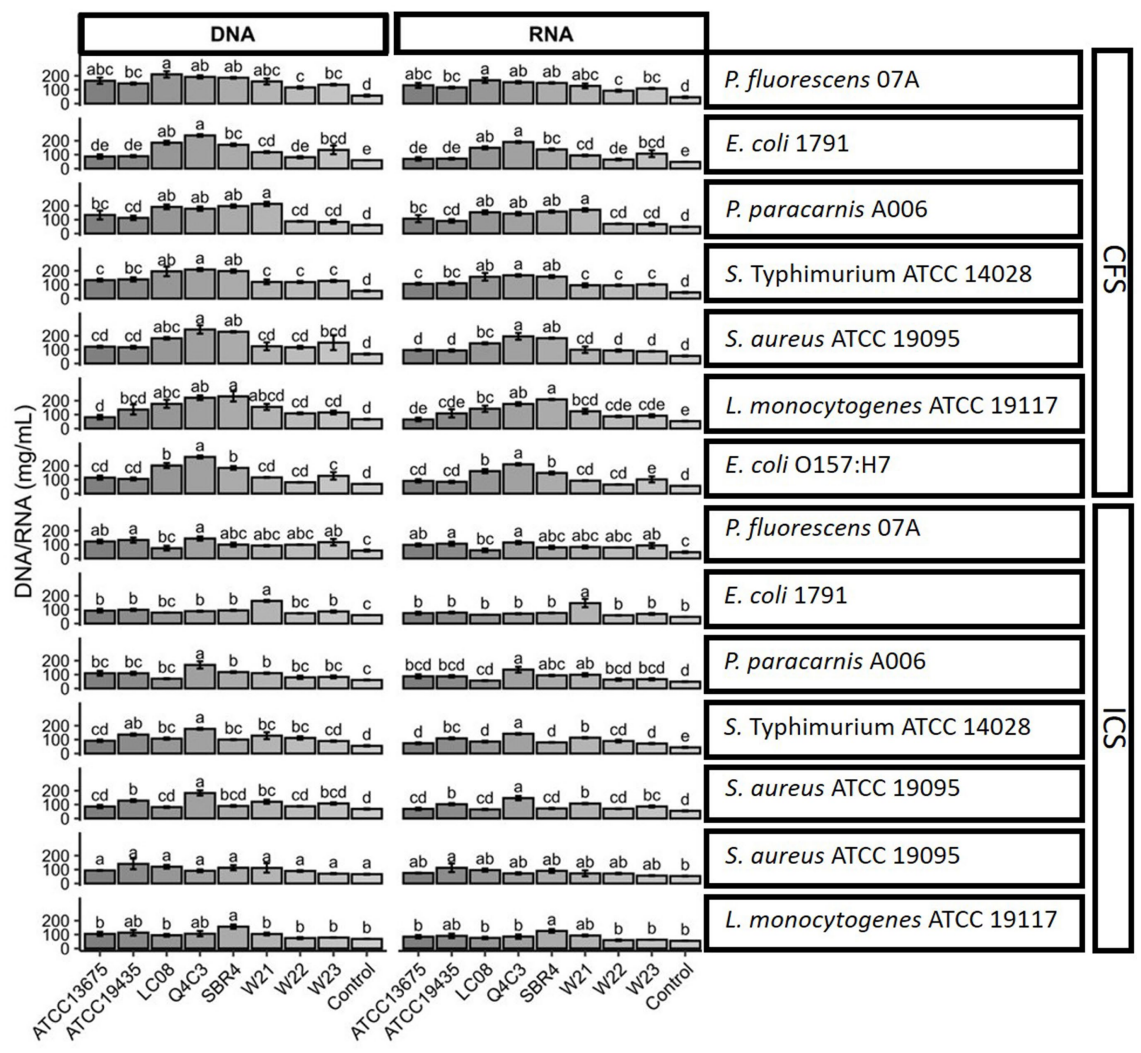
Bar chart illustrating cellular leakage in foodborne contaminants, quantified through DNA and RNA release after treatment with extracts of *Lc. lactis* subsp. *lactis* biovar diacetylactis ATCC 13675, *Lc. lactis* subsp. *lactis* ATCC 19435, *Lc. lactis* subsp. *lactis* Lc08, *Lpb. plantarum* Q4C3, *Lc. lactis* subsp. *lactis* biovar diacetylactis SBR4, *W. cibaria* W21, *W. cibaria* W22, and *W. viridescens* W23. The letters indicate statistically significant differences between each lactic acid bacteria, based on Tukey’s test (*p* < 0.05).

Cell leakage analysis revealed that CFS treatments resulted in higher extracellular nucleic acid levels than ICS treatments across all evaluated strains (Figure 5). Among the extracts, those from *Lc. lactis* subsp. *lactis* biovar diacetylactis SBR4, *Lpb. plantarum* Q4C3, *W. cibaria* W21, and *W. viridescens* W23 produced the highest leakage values.

### 3.4 Time-kill assay

For time–kill assessment, *P. fluorescens* 07A and *L. monocytogenes* were selected as representative Gram-negative and Gram-positive species. Extracts from the four LAB strains that produced the highest leakage values were included in this assay.

**Figure 6:**
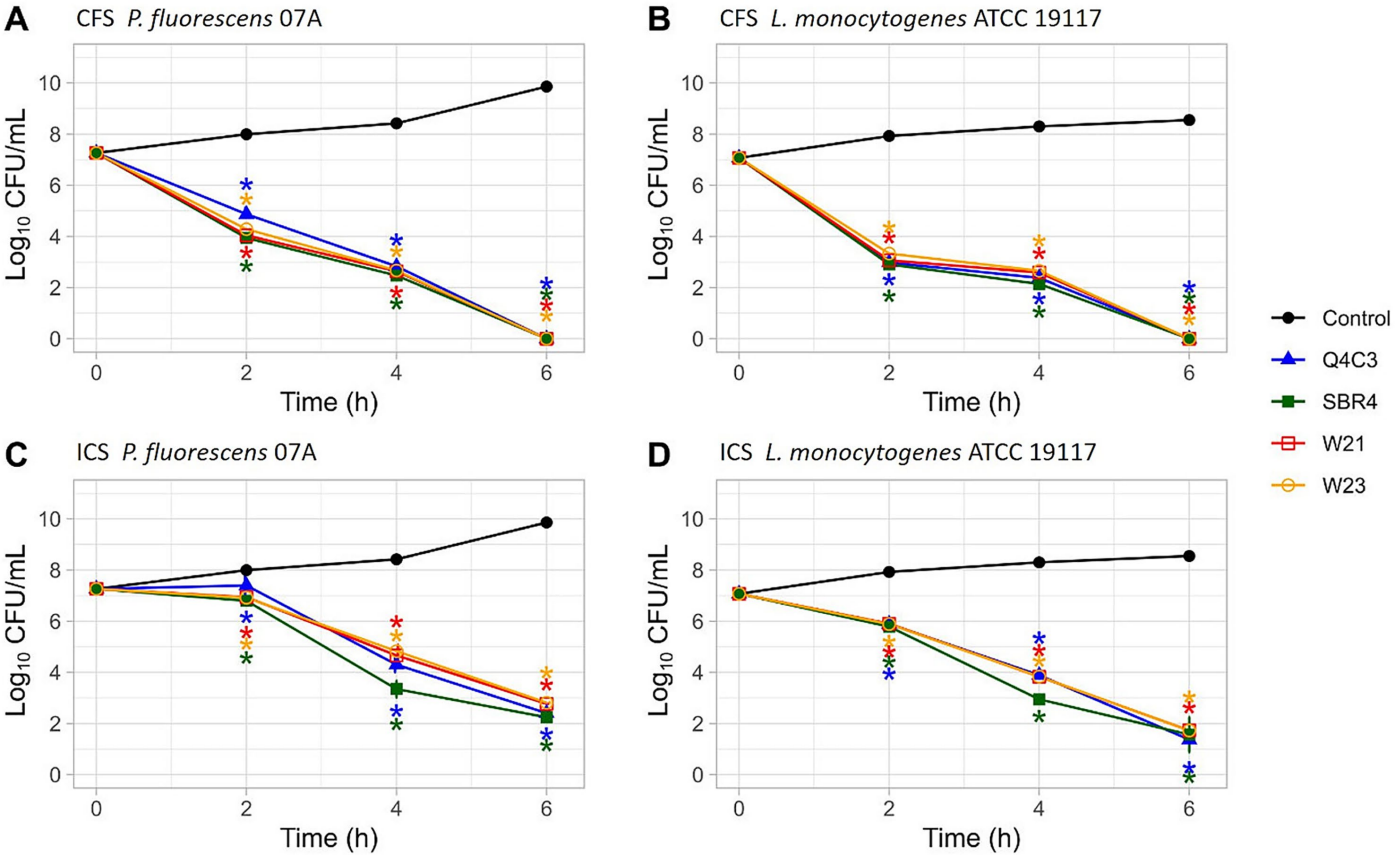
Time–kill curves of *P. fluorescens* 07A (**A**: CFS, **C**: ICS) and *L. monocytogenes* ATCC19117 (**B**: CFS, **D**: ICS) treated with four LAB-derived extracts (*Lpb. plantarum* Q4C3, *Lc. lactis* subsp. *lactis* biovar diacetylactis SBR4, *Lc. lactis* subsp. *lactis* Lc08, *W. cibaria* W21, *W. viridescens* W23) compared with the untreated control. Panels A and B correspond to cell-free supernatants (CFS), while panels C and D correspond to inactive cells plus cell-free supernatant (ICS). Bacterial counts (log₁₀ CFU/mL) were determined at 0, 2, 4, and 6 h of incubation. The asterisks indicate statistically significant differences between each lactic acid bacteria, based on Tukey’s test (*p* < 0.05).

CFS treatments resulted in reductions of more than 3 log CFU/mL within 4 h, while ICS tretments produced reductions of approximately 2 log CFU/mL over the same time (Figure 6). For CFS, no viable cells were detected after 6 h. For ICS, viable *P. fluorescens* 07A cells were detected at 4 h, followed by a marked reduction at 6 h. Therefore, 4 h was selected as the contact time for ICS and CFS treatments for next assays.

### 3.5 Organic acid quantification assay

The organic acid profile varied considerably among the eight tested LAB-derived extracts (Table 3). Detected organic acids included lactic, formic, acetic, citric, succinic, propionic, isobutyric, butyric, isovaleric, and valeric acids. *Lc. lactis* subsp. *lactis* biovar diacetylactis ATCC 13675 did not produce formic acid and showed the highest acetic acid levels. This strain and *Lpb. plantarum* Q4C3 did not produce citric acid. Isovaleric acid was detected only in *Lc. lactis* subsp. *lactis* biovar diacetylactis SBR4 and *Lc. lactis* subsp. *lactis* Lc08. Acid concentrations were lower in ICS compared with CFS for all strains.

**Table 3:** Concentrations (mmol/L) of organic acids detected by high-performance liquid chromatography (HPLC) in cell-free supernatants (CFS) and in inactive cells plus supernatant (ICS) derived from LAB.

LAB	Latic	Citric	Succinic	Formic	Acetic	Propionic	Isobutiric	Butiric	Isovaleric	Valeric
CFS										
Q4C3	189.657	0.000	4.124	94.381	90.179	13.167	0.511	28.253	0.000	0.071
SBR4	256.962	10.365	2.252	101.279	72.974	0.270	0.533	2.871	0.083	0.038
LC08	279.431	12.502	3.077	79.968	88.306	1.734	0.944	0.205	0.707	0.160
ATCC 13675	169.543	0.000	2.823	0. 000	137.138	3.358	1.089	0.504	0.000	0.000
ATCC 19435	191.744	11.328	3.947	68.389	78.583	13.247	0.883	26.950	0.000	0.110
W21	164.604	11.099	3.883	60.599	79.309	14.017	0.897	33.268	0.000	0.135
W22	183.114	3.212	6.550	83.348	94.540	15.415	1.149	34.518	0.000	0.140
W23	164.000	11.162	4.050	71.696	79.494	14.668	1.378	34.466	0.000	0.000
ICS										
Q4C3	167.664	0.000	3.086	73.892	92.033	12.350	0.665	32.878	0.000	0.100
SBR4	187.829	10.247	0.938	77.992	69.889	0.211	0.483	3.124	0.000	0.119
LC08	181.668	11.212	1.130	70.564	77.135	0.879	0.657	0.204	0.381	0.130
ATCC 13675	158.351	0.000	3.297	0.000	90.769	20.496	1.283	0.464	0.000	0.135
ATCC 19435	188.513	11.299	4.197	67.659	79.593	15.140	1.284	24.378	0.000	0.114
W21	158.572	10.614	3.892	61.267	77.673	14.458	1.229	31.670	0.000	0.047
W22	154.039	10.148	3.409	60.374	74.436	12.518	0.844	33.325	0.000	0.099
W23	157.988	10.578	3.906	68.385	77.738	0.151	1.308	31.509	0.000	0.139

*Lpb. plantarum* Q4C3, *Lc. lactis* subsp. *lactis* biovar diacetylactis SBR4, *Lc. lactis* subsp. *lactis* Lc08, *Lc. lactis* subsp. *lactis* biovar diacetylactis ATCC 13675, *Lc. lactis* subsp. *lactis* ATCC 19435, *W. cibaria* W21, *W. cibaria* W22, *W. viridescens* W23.

### 3.6 SEM

**Figure 7:**
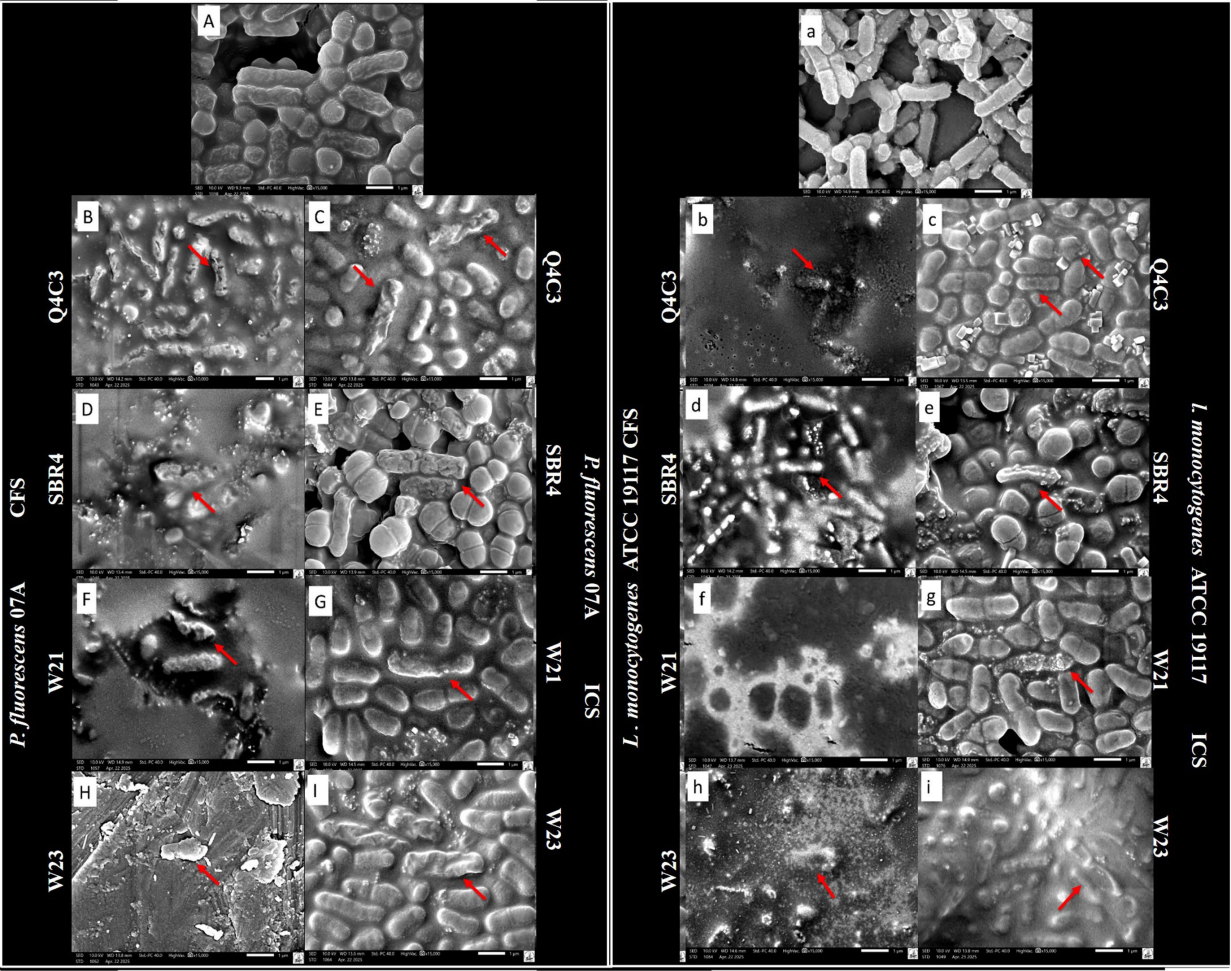
SEM images of *Pseudomonas fluorescens* 07A cells (**A**: Control, **B, C, D, E, F, G, H, I**) and *Listeria monocytogenes* ATCC 19117 cells (**a**: Control, **b, c, d, e, f, g, h, i**) treated with LAB-derived extract at 1×MBC from *Lpb. plantarum* Q4C3 (B/b: CFS; C/c: ICS), *Lc. lactis* subsp. *lactis* biovar diacetylactis SBR4 (D/d: CFS; E/e: ICS), *W. cibaria* W21 (F/f: CFS; G/g: ICS) and *W. viridescens* 23 derived extracts (H/h: CFS; I/i: ICS) for 4 h. The red arrows indicate irregular and collapsed surfaces, fissures, and deformations, which are indicative of membrane damage and possible cell lysis.

SEM images revealed structural alterations in bacterial cells following exposure to LAB-derived extracts (Figure 7). For *P. fluorescens* 07A, CFS-treated cells showed more extensive deformation than ICS-treated cells. Similar patterns were observed for *L. monocytogenes*. Control cells of both species maintained intact morphology, whereas treated cells exhibited varied degrees of surface irregularity, including depressions and distortions. Morphological changes were consistently more pronounced in cells treated with CFS compared with ICS.

## 4 Discussion

In this study, LAB-derived extracts exhibited marked antimicrobial and antibiofilm activities against foodborne pathogens and spoilage microorganisms relevant to dairy systems. However, the magnitude and spectrum of inhibition were clearly strain dependent. In agar diffusion assay, *L. monocytogenes* ATCC 19117 and *P. fluorescens* were among the most susceptible targets, but inhibition patterns varied considerably across the 15 LAB strains (Figure 1), indicating that antagonistic effects are strain specific rather than uniform at the species level. The inhibition observed in agar assay reflects the direct interactions with the solid medium, where metabolite diffusion and localized competition play a key role, and may differ from liquid co-culture systems, in which nutrient availability, mixing, and metabolite dispersion influence the extent of antagonism (Selegato and Castro-Gamboa, 2023).

A consistent finding across assays was the superior performance of non-neutralized extracts (CFS and ICS) compared with their neutralized counterparts (CFS-N and ICS-N), both in terms of MIC values and antibiofilm activity. This pattern supports the central role of organic acids in the antimicrobial action of LAB-derived extracts. Partial or total loss of activity, following pH neutralization, as observed for several strains, reinforces this interpretation and agrees with reports in which non-neutralized supernatants from *Lpb. plantarum* exhibited strong antibacterial activity that was abolished upon neutralization (Grigore-Gurgu et al., 2024). In this study, *Lpb. plantarum* Q4C3 stood out not only for its antibacterial potential but also corroborates previous work in which this strain ranked among the most effective LAB against filamentous fungi such as *Aspergillus niger* IOC 207 and *Penicillium chrysogenum* IOC 132 (Souza et al., 2023), highlighting its versatility as a biocontrol agent.

The organic acid profiles obtained for selected strains help to explain these observer results in this studye observations. Lactic, acetic and propionic acids were detected in the highest concentrations, with additional contributions from formic, citric, succinic, butyric and valeric acids. The transition from CFS to ICS was accompanied by a general decrease in organic acid concentrations and a subtle increase in pH, suggesting partial neutralization of acids due to the release of intracellular fluid following cell lysis. Weak acids display greater antimicrobial activity at low pH, and acetic and propionic acids are typically more potent inhibitors than lactic acid because of their higher pK_a_ values, which favor a higher proportion of undissociated molecules in acidic environments (Lahtinen et al., 2011; Wang et al., 2014; Ray and Sandine, 2019). In mixed acid systems, lactic acid contributes predominantly to pH reduction, whereas acetic and propionic acids act as the main antimicrobial components, often exhibiting synergistic effects (Zavišić et al., 2024). The composition and concentration of these acids in CFS and ICS therefore strongly influence the observed inhibitory profiles.

The biological relevance of the organic acid profiles obtained by HPLC can be further interpreted by comparison with inhibitory concentrations reported in food-relevant conditions. Wemmenhove et al. (2016) determined the MIC of undissociated lactic, acetic, citric, and propionic acids against *L. monocytogenes* at cheese-relevant pH conditions (5.2–5.6), reporting average MIC of approximately 5 mM for lactic acid, 19 mM for acetic acid, and 11 mM for propionic acid. These values overlap with the organic acid concentrations detected in the most active LAB-derived extracts evaluated in the present study, supporting the biological plausibility of the observed antimicrobial effects in dairy matrices. In addition, salt content and other intrinsic factors in cheese matrices may further reduce the effective MICs of organic acids, thereby enhancing their inhibitory potential.

However, in complex food environments such as the moisture phase of cheeses, dilution effects and matrix buffering can decrease the proportion of undissociated acids available to exert antimicrobial activity. Factors including pH, water activity, ionic strength, and the presence of competitive microflora can modulate pathogen inhibition and should be considered when extrapolating *in vitro* efficacy to real food systems (Engstrom et al., 2020; Falih et al., 2024). Together, these considerations indicate that while organic acid concentrations measured in LAB-derived extracts are within inhibitory ranges reported for dairy-relevant conditions, their effective antimicrobial performance in foods will depend on matrix-specific interactions.

Although pH-neutralization experiments provide valuable insight into acid-mediated antimicrobial mechanisms, they do not exclude the contribution of pH-sensitive bacteriocins or other metabolites. Several antimicrobial peptides produced by LAB are known to be more active and stable under acidic conditions, while exhibiting reduced activity or structural destabilization at neutral or alkaline pH due to changes in ionization state and partial peptide denaturation (Liang et al., 2025). A similar pH-dependent loss of activity has been reported for nisin, which shows significantly reduced inhibitory effects as pH increases (Zhang et al., 2016). Therefore, the reduction in antimicrobial efficacy observed after neutralization may reflect not only the loss of organic acid activity but also diminished functionality of bacteriocins and other acid-dependent metabolites.

Consistent with this interpretation, residual antimicrobial activity detected in some neutralized extracts indicates the contribution of additional acid-independent metabolites. In the present study, *Lc. lactis* subsp. *lactis* Lc08 retained antimicrobial activity after neutralization, in agreement with previous reports describing the production of antagonistic compounds that remain stable over a wide pH range and are only inactivated after autoclaving (Perin et al., 2013), in this study retained antimicrobial activity after neutralization, consistent with the presence of acid-independent compounds. Similarly, *Lc. lactis* subsp. *lactis* bv. diacetylactis SBR4, characterized as a nisin Z producer with high solubility and diffusibility at neutral pH (Fusieger et al., 2020b), showed consistent behavior with its known characteristics. Together, these results support a model in which organic acids provide a basal antimicrobial effect that can be further enhanced by bacteriocins, in line with previous evidence that the co-application of acids and bacteriocins results in synergistic inhibition of pathogens and spoilers (Yu et al., 2023).

*Weissella* strains also emerged as promising candidates for biocontrol in dairy environments. In agreement with previous studies showing that *W. cibaria* W21 and related strains inhibit a range of foodborne (Dey et al., 2019; Teixeira et al., 2021), this results demonstrate that *W. cibaria* W21, *W. cibaria* W22 and *W. viridescens* W23 combine relevant antimicrobial and antibiofilm activities. The fact that some *Weissella*-derived extracts retained partial activity after neutralization suggests the production of additional bioactive metabolites beyond organic acids, reinforcing the potential of this genus as a source of bacteriocins and extracts preparations.

Although *Weissella* strains have demonstrated relevant antimicrobial and antibiofilm potential, their application as live cultures should be approached with caution. This genus lacks Qualified Presumption of Safety (QPS) status according to the EFSA and GRAS recognition by the FDA, and safety has not been established for all strains (FDA, 2024; EFSA, 2026). This regulatory limitation highlights the need for strain-specific safety assessments prior to industrial use. In this context, the application of cell-free extracts derived from *Weissella* represents a safer alternative, allowing the exploitation of bioactive metabolites such as organic acids and bacteriocins, without the risks associated with administering live microrganisms (Fusco et al., 2023). The use of *Weissella* derived cell-free extracts or LAB-derived extracts strain-level antagonistic evaluation and supports the development of natural, safe, and industrially applicable biocontrol strategies.

Beyond safety considerations, an additional layer of complexity in biocontrol strategies is that antimicrobial efficacy against planktonic cells does not necessarily translate into effective biofilm inhibition. This discrepancy indicates that biofilm inhibition cannot be ascribed solely to reductions in viable cell numbers. As highlighted by Sharma et al. (2023), surface adhesion represents only the initial step of biofilm development; and compounds that directly interfere with exopolysaccharide synthesis, motility, or quorum sensing can markedly alter biofilm architecture. In this context, LAB-derived metabolites, such as hydrogen peroxide, oxygenated compounds, exopolysaccharides, bacteriocins, and fatty acids with surfactant properties, have been identified as key antibiofilm agents (Moradi et al., 2019; Rather et al., 2021). In this study, LAB-derived extracts exhibited strain-dependent antimicrobial and antibiofilm activities against both spoilage and pathogenic microorganisms, highlighting the diversity of antagonistic mechanisms. Z-score-based ranking and growth and biofilm inhibition data (Figure 2) confirmed that only a subset of LAB strains combines consistently high performance across both phenotypes, underscoring the need for strain-level selection when designing biocontrol strategies.

Within this context, the physical composition of LAB-derived preparations, particularly the presence of cellular debris in ICS, emerges as a critical factor influencing antimicrobial and antibiofilm performance. The presence of cellular debris in some treatments was associated with a reduction in antimicrobial potential, particularly for ICS. One plausible explanation is that cell remnants, extracellular polymeric substances, proteins, lipids, and nucleic acids may form a physical barrier around target cells, partially shielding them from diffusible antimicrobial metabolites (Zhao et al., 2017). Similar mechanisms have been proposed to explain decreased antimicrobial activity in non-filtered preparations or in systems with high organic load (Ostad et al., 2009). Conversely, other studies suggest that inactivated LAB cells can reduce pathogen adhesion by forming a physical layer on the surface, thereby limiting colonization (Singh et al., 2017; Zielińska et al., 2021). These apparently conflicting observations highlight that the role of cellular debris is context dependent and influenced by factors such as matrix composition, microbial species and the relative contribution of diffusible versus cell-associated components. In our experimental conditions, filtration and removal of cellular remnants improved antimicrobial performance, which is consistent with the higher activity observed for CFS compared with ICS. In pratical applications, the behavior of LAB-derived extracts in real food systems is influenced by the surrounding matrix. Organic matter can modulate acid efficacy, with some components reducing and others enhancing antimicrobial effects, depending on the type of acid and the food environment (Guan and Liu, 2020; Lund et al., 2020).

In addition to matrix-related effects, intrinsic structural differences between target microorganisms also influence the efficacy of LAB-derived extracts. A notable outcome was the ability of CFS to inhibit both Gram-positive and Gram-negative bacteria. While bacteriocins are generally more active against Gram-positive organisms because of the protective outer membrane in Gram-negative bacteria (Darbandi et al., 2022), the combination of organic acids and other metabolites present in CFS may compromise this barrier by increasing membrane permeability. Previous studies have shown that organic acids and CFS from LAB can induce leakage of K^+^ ions, nucleic acids and proteins, reflecting direct damage to the cytoplasmic membrane (Bajpai et al., 2016; Sun et al., 2024; Wang et al., 2023). MIC and time–kill data, together with leakage assays, support a mechanism in which acids and associated metabolites facilitate access of bioactive compounds to the cell membrane, broadening the inhibitory spectrum to include Gram-negative spoilers such as *Pseudomonas* spp.

Cell leakage and SEM analyses provided further insight into the mode of action of LAB-derived extracts. CFS treatments consistently resulted in higher extracellular nucleic acid levels than ICS, and SEM images revealed pronounced morphological alterations in *P. fluorescens* and *L. monocytogenes* cells exposed to CFS, including surface collapse, wrinkling and structural distortion. These findings are in line with previous studies reporting membrane damage, cell swelling, vacuole formation and leakage of intracellular components in bacteria treated with LAB CFS (Abou Elez et al., 2023; Wang et al., 2023; Sun et al., 2024). Taken together, these observations support the notion that LAB-derived metabolites interact with membrane phospholipids and proteins, alter membrane fluidity, promote pore formation and ultimately lead to leakage and cell death (Anumudu et al., 2024; Che et al., 2024; Rahman et al., 2025; Banicod et al., 2025).

The membrane damage and leakage effects observed by SEM and nucleic acid release assays are further reflected in the rapid bactericidal kinetics revealed by the time–kill experiments. The time-kill assays highlight not only the bactericidal capacity of the extracts but also the speed of their action. For *L. monocytogenes*, CFS caused rapid reductions in viable counts, whereas *P. fluorescens* required slightly longer exposure for comparable effects. ICS required longer contact times than CFS in both cases. When compared with previous work in which LAB CFS required longer exposures or higher multiples of the MIC to achieve modest log reductions (Kho et al., 2024; Rotta et al., 2025; Liang et al., 2025), the extracts evaluated here displayed faster and more pronounced bactericidal activity. This enhanced efficacy may reflect the combined effects of a favorable organic acid profile and additional diffusible metabolites, as suggested by both the MIC/MBC results and the leakage and SEM data.

## 5 Conclusion

This present work demonstrated that LAB-derived extracts, particularly CFS from *Lpb. plantarum* Q4C3, *Lc. lactis* subsp. *lactis* biovar diacetylactis SBR4, *W. cibaria* W21, and *W. viridescens* W23, combine rapid bactericidal activity, antibiofilm effects, and a broad inhibitory spectrum against dairy-related contaminants. By systematically comparing multiple LAB strains and extract types, this study identified the most effective strain and extract combinations for potential industrial application. These findings support the use of these extracts as natural, fast-acting biocontrol agents to improve the microbial safety and quality of dairy products, while also providing a framework for future studies in real food matrices and pilot-scale applications.

## Data Availability

The original contributions presented in the study are included in the article/Supplementary material, further inquiries can be directed to the corresponding authors.

## References

[ref1] Abou Elez R. M. M. Elsohaby I. al-Mohammadi A. R. Seliem M. Tahoun A. B. M. B. Abousaty A. I. . (2023). Antibacterial and anti-biofilm activities of probiotic *Lactobacillus plantarum* against *Listeria monocytogenes* isolated from milk, chicken and pregnant women. Front. Microbiol. 14:1201201. doi: 10.3389/fmicb.2023.1201201, 37538844 PMC10394229

[ref2] Anumudu C. K. Miri T. Onyeaka H. (2024). Multifunctional applications of lactic acid bacteria: enhancing the safety, quality, and nutritional value of fermented foods and beverages. Foods 13:3714. doi: 10.3390/foods13233714 39682785 PMC11640447

[ref3] Arena M. P. Silvain A. Normanno G. Grieco F. Drider D. Spano G. . (2016). Use of *Lactobacillus plantarum* strains as a bio-control strategy against food-borne pathogenic microorganisms. Front. Microbiol. 7:464. doi: 10.3389/fmicb.2016.00464, 27148172 PMC4829616

[ref4] Ashrafudoulla M. Park J. Toushik S. H. Shaila S. Ha A. J.-w. Rahman M. A. . (2024). Synergistic mechanism of UV-C and postbiotic of *Leuconostoc mesenteroides* (J.27) combination to eradicate *Salmonella Thompson* biofilm in the poultry industry. Food Control 164:110607. doi: 10.1016/j.foodcont.2024.110607

[ref5] Bajpai V. K. Han J. H. Rather I. A. Park C. Lim J. Paek W. K. . (2016). Characterization and antibacterial potential of lactic acid bacterium *Pediococcus pentosaceus* 4I1 isolated from freshwater fish *Zacco koreanus*. Front. Microbiol. 7:2037. doi: 10.3389/fmicb.2016.02037, 28066360 PMC5167689

[ref6] Banicod R. J. S. Tabassum N. Javaid A. Kim Y. M. Khan F. (2025). Lactic acid bacteria–derived secondary metabolites: emerging natural alternatives for food preservation. Probiotics Antimicrob. Proteins, 1–38. doi: 10.1007/s12602-025-10672-6 40751876

[ref7] Che J. Shi J. Fang C. Zeng X. Wu Z. Du Q. . (2024). Elimination of pathogen biofilms via postbiotics from lactic acid bacteria: a promising method in food and biomedicine. Microorganisms 12:704. doi: 10.3390/microorganisms12040704, 38674648 PMC11051744

[ref8] Choi D. Bedale W. Chetty S. Yu J. H. (2024). Comprehensive review of clean-label antimicrobials used in dairy products. Compr. Rev. Food Sci. Food Saf. 23:e13263. doi: 10.1111/1541-4337.13263, 38284580

[ref9] Darbandi A. Asadi A. Mahdizade Ari M. Ohadi E. Talebi M. Halaj Zadeh M. . (2022). Bacteriocins: properties and potential use as antimicrobials. J. Clin. Lab. Anal. 36:e24093. doi: 10.1002/jcla.24093, 34851542 PMC8761470

[ref10] Dey D. K. Koo B. G. Sharma C. Kang S. C. (2019). Characterization of *Weissella confusa* DD_A7 isolated from kimchi. LWT 111, 663–672. doi: 10.1016/j.lwt.2019.05.089

[ref11] Du J. Zhang J. Zhang D. Zhou Y. Wu P. Ding W. . (2022). Background filtering of clinical metagenomic sequencing with a library concentration-normalized model. Microbiol. Spectrum 10, e01779–e01722. doi: 10.1128/spectrum.01779-22, 36135379 PMC9603461

[ref12] EFSA (2026). Update of the list of qualified presumption of safety (QPS) recommended microbiological agents intentionally added to food or feed as notified to EFSA 23: suitability of taxonomic units notified to EFSA until September 2025. Parma: EFSA. 10.2903/j.efsa.2026.9824 PMC12824592 41583339

[ref13] Engstrom S. K. Cheng C. Seman D. Glass K. A. (2020). Growth of *Listeria monocytogenes* in a model high-moisture cheese on the basis of pH, moisture, and acid type. J. Food Prot. 83, 1335–1344. doi: 10.4315/JFP-20-069, 32221553

[ref14] Falih M. A. Altemimi A. B. Alkaisy Q. H. Awlqadr F. H. Abedelmaksoud T. G. Amjadi S. . (2024). Enhancing safety and quality in the global cheese industry: a review of innovative preservation techniques. Heliyon 10:23. doi: 10.1016/j.heliyon.2024.e40459, 39654744 PMC11625285

[ref15] FDA (2024). Microorganisms & Microbial-Derived Ingredients Used in food (partial list). Available online at: https://www.fda.gov/food/generally-recognized-safe-gras/microorganisms-microbial-derived-ingredients-used-food-partial-list (Accessed February 5, 2025).

[ref16] Fusco V. Chieffi D. Fanelli F. Logrieco A. F. Cho G. S. Kabisch J. . (2020). Microbial quality and safety of milk and milk products in the 21st century. Compr. Rev. Food Sci. Food Saf. 19, 2013–2049. doi: 10.1111/1541-4337.12568, 33337106

[ref17] Fusco V. Chieffi D. Fanelli F. Montemurro M. Rizzello C. G. Franz C. M. (2023). The *Weissella* and *Periweissella* genera: up-to-date taxonomy, ecology, safety, biotechnological, and probiotic potential. Front. Microbiol. 14:1289937. doi: 10.3389/fmicb.2023.1289937, 38169702 PMC10758620

[ref18] Fusieger A. Martins M. C. F. de Freitas R. Nero L. A. de Carvalho A. F. (2020a). Technological properties of *Lactococcus lactis* subsp. *lactis* bv. *Diacetylactis* obtained from dairy and non-dairy niches. Braz. J. Microbiol. 51, 313–321. doi: 10.1007/s42770-019-00182-3, 31734902 PMC7058814

[ref19] Fusieger A. Perin L. M. Teixeira C. G. de Carvalho A. F. Nero L. (2020b). The ability of *Lactococcus lactis* subsp. *lactis* bv. *Diacetylactis* strains in nisin production. Antonie Van Leeuwenhoek 5, 651–662. doi: 10.1007/s10482-019-01373-6 31838601

[ref20] Grigore-Gurgu L. Cotârleț M. Pihurov M. Păcularu-Burada B. Vasile A. M. Enachi E. . (2024). *Lactiplantibacillus plantarum* and *Lactiplantibacillus paraplantarum* postbiotics: assessment of the biotic-derived metabolites with cytocompatibility and antitumoral potential. Food Biosci. 59:103863. doi: 10.1016/j.fbio.2024.103863

[ref21] Guan N. Liu L. (2020). Microbial response to acid stress: mechanisms and applications. Appl. Microbiol. Biotechnol. 104, 51–65. doi: 10.1007/s00253-019-10226-1, 31773206 PMC6942593

[ref22] İncili G. K. Karatepe P. Akgöl M. Kaya B. Kanmaz H. Hayaloğlu A. A. (2021). Characterization of *Pediococcus acidilactici* postbiotic and impact of postbiotic-fortified chitosan coating on the microbial and chemical quality of chicken breast fillets. Int. J. Biol. Macromol. 184, 429–437. doi: 10.1016/j.ijbiomac.2021.06.106, 34166693

[ref23] Jayarao B. M. Henning D. R. (2001). Prevalence of foodborne pathogens in bulk tank milk. J. Dairy Sci. 84, 2157–2162. doi: 10.3168/jds.S0022-0302(01)74661-9, 11699446

[ref24] Jeong J. H. Park S. Jang M. Kim K.-s. (2024). Evaluating the antagonistic activity of lactic acid bacteria in cadaverine production by *Vibrio* strains during co-culture. Fermentation 10:356. doi: 10.3390/fermentation10070356

[ref25] Jiang X. Jiang C. Yu T. Jiang X. Kang R. Ren S. . (2022). Phenyllactic acid application to control *Listeria monocytogenes* biofilms and its growth in milk and spiced beef. Int. J. Food Microbiol. 381:109910. doi: 10.1016/j.ijfoodmicro.2022.109910, 36063683

[ref26] Karabacak Aydin E. G. Mohammed S. Con A. H. (2026). Postbiotics in the food industry: applications, delivery systems, and future perspectives. Arch. Microbiol. 208:38. doi: 10.1007/s00203-025-04577-9, 41284006

[ref27] Kho K. Kadar A. D. Bani M. D. Pramanda I. T. Martin L. Chrisdianto M. . (2024). The potential of *Pediococcus acidilactici* cell-free supernatant as a preservative in food packaging materials. Foods 13:644. doi: 10.3390/foods13050644, 38472756 PMC10930656

[ref28] Kim Y. K. Roy P. K. Ashrafudoulla M. Nahar S. Toushik S. H. Hossain M. I. . (2022). Antibiofilm effects of quercetin against *Salmonella enterica* biofilm formation and virulence, stress response, and quorum-sensing gene expression. Food Control 137:108964. doi: 10.1016/j.foodcont.2022.108964

[ref29] Kousta M. Mataragas M. Skandamis P. Drosinos E. H. (2010). Prevalence and sources of cheese contamination with pathogens at farm and processing levels. Food Control 21, 805–815. doi: 10.1016/j.foodcont.2009.11.015

[ref30] Kumar H. Franzetti L. Kaushal A. Kumar D. (2019). *Pseudomonas fluorescens*: a potential food spoiler and challenges and advances in its detection. Ann. Microbiol. 69, 873–883. doi: 10.1007/s13213-019-01501-7

[ref31] Lahtinen S. Ouwehand A. C. Salminen S. Wright A. V. (2011). Lactic acid bacteria: microbiological and functional aspects. Boca Raton, FL: CRC Press.

[ref32] Liang Q. Liu Z. Liang Z. Fu X. Li D. Zhu C. . (2025). Current challenges and development strategies of bacteriocins produced by lactic acid bacteria applied in the food industry. Compr. Rev. Food Sci. Food Saf. 24:e70038. doi: 10.1111/1541-4337.70038I 39674838

[ref33] Liang M. Wang H. Zhou Z. Huang Y. Suo H. (2025). Antibacterial mechanism of *Lactiplantibacillus plantarum* SHY96 cell-free supernatant against *Listeria monocytogenes* revealed by metabolomics and potential application on chicken breast meat preservation. Food Chem. X 25:102078. doi: 10.1016/j.fochx.2024.102078, 39758074 PMC11699396

[ref34] Lund P. A. de Biase D. Liran O. Scheler O. Mira N. P. Cetecioglu Z. . (2020). Understanding how microorganisms respond to acid pH is central to their control and successful exploitation. Front. Microbiol. 11:556140. doi: 10.3389/fmicb.2020.556140, 33117305 PMC7553086

[ref35] Mantovam V. B. Dos Santos D. F. Giola Junior L. C. Landgraf M. Pinto U. M. Todorov S. D. (2025). *Listeria monocytogenes*, Salmonella spp., and *Staphylococcus aureus*: threats to the food industry and public health. Foodborne Pathog. Dis. 22, 809–824. doi: 10.1089/fpd.2024.0124 39761068

[ref36] Martin N. H. Evanowski R. L. Wiedmann M. (2023). Invited review: redefining raw milk quality - evaluation of raw milk microbiological parameters to ensure high-quality processed dairy products. J. Dairy Sci. 106, 1502–1517. doi: 10.3168/jds.2022-22416, 36631323

[ref37] Martin N. H. Trmčić A. Hsieh T. H. Boor K. J. Wiedmann M. (2016). The evolving role of coliforms as indicators of unhygienic processing conditions in dairy foods. Front. Microbiol. 7:1549. doi: 10.3389/fmicb.2016.01549, 27746769 PMC5043024

[ref38] Moradi M. Kousheh S. A. Almasi H. Alizadeh A. Guimarães J. T. Yılmaz N. . (2020). Postbiotics produced by lactic acid bacteria: the next frontier in food safety. Compr. Rev. Food Sci. Food Saf. 19, 3390–3415. doi: 10.1111/1541-4337.12613, 33337065

[ref39] Moradi M. Mardani K. Tajik H. (2019). Characterization and application of postbiotics of *Lactobacillus* spp. on *Listeria monocytogenes* in vitro and in food models. LWT 111, 457–464. doi: 10.1016/j.lwt.2019.05.072

[ref40] Morton R. D. (2001). Aerobic plate count, compendium of methods for the microbiological examination of foods, vol. 4. Washington DC: American Public Health, 63–67.

[ref41] Oliver S. P. Jayarao B. M. Almeida R. A. (2005). Foodborne pathogens in milk and the dairy farm environment: food safety and public health implications. Foodbourne Pathogens Disease 2, 115–129. doi: 10.1089/fpd.2005.2.115, 15992306

[ref42] Ostad S. N. Salarian A. A. Ghahramani M. H. Fazeli M. R. Samadi N. Jamalifar H. (2009). Live and heat-inactivated lactobacilli from feces inhibit *Salmonella Typhi* and *Escherichia coli* adherence to Caco-2 cells. Folia Microbiol. 54, 157–160. doi: 10.1007/s12223-009-0024-7, 19418255

[ref43] Perin L. M. Miranda R. O. Camargo A. C. Colombo M. Carvalho A. F. Nero L. A. (2013). Antimicrobial activity of the Nisin Z producer *Lactococcus lactis* subsp. *lactis* Lc08 against *Listeria monocytogenes* in skim milk. Arq. Bras. Med. Vet. Zootec. 65, 1554–1560. doi: 10.1590/S0102-09352013000500037

[ref44] Pinto C. L. O. (2004). Proteolytic psychrotrophic bacteria in bulk cooled raw milk intended for UHT milk production, thesis. Viçosa, MG: Federal University of Viçosa, 91.

[ref45] Rahman M. M. Sazili A. Q. Ahmad S. A. Khalil K. A. Ismail-Fitry M. R. Sarker M. S. K. (2025). Inhibitory efficacy, production dynamics, and characterization of postbiotics of lactic acid bacteria. BMC Microbiol. 25:485. doi: 10.1186/s12866-025-04123-z, 40764529 PMC12326657

[ref46] Raman J. Kim J. S. Choi K. R. Eun H. Yang D. Ko Y. J. . (2022). Application of lactic acid bacteria (LAB) in sustainable agriculture: advantages and limitations. Int. J. Mol. Sci. 23:7784. doi: 10.3390/ijms23147784, 35887142 PMC9322495

[ref47] Rather M. A. Gupta K. Mandal M. (2021). Microbial biofilm: formation, architecture, antibiotic resistance, and control strategies. Braz. J. Microbiol. 52, 1701–1718. doi: 10.1007/s42770-021-00624-x, 34558029 PMC8578483

[ref48] Ray B. Sandine W. E. (2019). Acetic, propionic, and lactic acids of starter culture bacteria as biopreservatives in Food biopreservatives of microbial origin. Boca Raton, FL, USA: CRC Press. 103–136.

[ref49] Rodrigues R. Rodrigues R. d. S. Machado S. G. Fernandes d. C. A. Nero L. A. (2021). *Pseudomonas* sp. as the causative agent of anomalous blue discoloration in Brazilian fresh soft cheese (Minas Frescal). Int. Dairy J. 117:105020. doi: 10.1016/j.idairyj.2021.105020

[ref50] Rotta I. S. Rezende S. D. D. C. Perini H. F. da Silva M. V. de Almeida F. A. Pinto U. M. . (2025). Exploring the potential of *Weissella paramesenteroides* UFTM 2.6.1 in disrupting quorum sensing and attenuating virulence in *Listeria monocytogenes*. Front. Microbiol. 16:1601203. doi: 10.3389/fmicb.2025.1601203, 40606152 PMC12213706

[ref51] Sadekuzzaman M. Mizan M. F. R. Kim H.-S. Yang S. Ha S.-D. (2018). Activity of thyme and tea tree essential oils against selected foodborne pathogens in biofilms on abiotic surfaces. LWT 89, 134–139. doi: 10.1016/j.lwt.2017.10.042

[ref52] Salminen S. Collado M. C. Endo A. Hill C. Lebeer S. Quigley E. M. . (2021). The international scientific Association of Probiotics and Prebiotics (ISAPP) consensus statement on the definition and scope of postbiotics. Nat. Rev. Gastroenterol. Hepatol. 19, 551–667. doi: 10.1038/s41575-021-00440-6, 33948025 PMC8387231

[ref53] Selegato D. M. Castro-Gamboa I. (2023). Enhancing chemical and biological diversity by co-cultivation. Front. Microbiol. 14:1117559. doi: 10.3389/fmicb.2023.1117559, 36819067 PMC9928954

[ref54] Sharafi H. Divsalar E. Rezaei Z. Liu S.-Q. Moradi M. (2023). The potential of postbiotics as a novel approach in food packaging and biopreservation: a systematic review of the latest developments. Crit. Rev. Food Sci. Nutr. 64, 12524–12554. doi: 10.1080/10408398.2023.2253909, 37667831

[ref55] Sharma S. Mohler J. Mahajan S. D. Schwartz S. A. Bruggemann L. Aalinkeel R. (2023). Microbial biofilm: a review on formation, infection, antibiotic resistance, control measures, and innovative treatment. Microorganisms 11:1614. doi: 10.3390/microorganisms11061614, 37375116 PMC10305407

[ref56] Singh T. P. Kaur G. Kapila S. Malik R. K. (2017). Antagonistic activity of *Lactobacillus reuteri* strains on the adhesion characteristics of selected pathogens. Front. Microbiol. 8:486. doi: 10.3389/fmicb.2017.00486, 28377765 PMC5359300

[ref57] Snyder A. B. Martin N. Wiedmann M. (2024). Microbial food spoilage: impact, causative agents and control strategies. Nat. Rev. Microbiol. 22, 528–542. doi: 10.1038/s41579-024-01037-x, 38570695

[ref58] Soltani S. Biron E. Ben Said L. Subirade M. Fliss I. (2022). Bacteriocin-based synergetic consortia: a promising strategy to enhance antimicrobial activity and broaden the spectrum of inhibition. Microbiol. Spectr. 10:e00406-21. doi: 10.1128/spectrum.00406-21, 35170996 PMC8849083

[ref59] Souza L. V. da Rodrigues Silva R. Falqueto A. Fusieger A. Martins E. Caggia C. . (2023). Evaluation of antifungal activity of lactic acid bacteria against fungi in simulated cheese matrix. LWT 182:114773. doi: 10.1016/j.lwt.2023.114773

[ref60] Sulistyani N. Mahfudh N. Mawardi R. H. Zakaria Z. A. (2023). Cell leakage mechanism and time kill studies on *Staphylococcus aureus* after exposure to ethanol leaf extract of *Muntingia calabura* L. Trop. J. Pharm. Res. 22, 355–362. doi: 10.4314/tjpr.v22i2.19

[ref61] Sun Y. Wei T. Ma T. Fan Z. Song J. (2024). *Dellaglioa algida* cell-free supernatant inhibits *Pseudomonas fluorescence* and *Pseudomonas fragi* by destroying cell membranes. Foods 13:2986. doi: 10.3390/foods13182986, 39335914 PMC11431788

[ref62] Teixeira C. G. Fusieger A. Martins E. Rosângela d. F. Vakarelova M. Nero L. A. . (2021). Biodiversity and technological features of *Weissella* isolates obtained from Brazilian artisanal cheese-producing regions. LWT 147:111474. doi: 10.1016/j.lwt.2021.111474

[ref63] Teneva D. Denev P. (2023). Biologically active compounds from probiotic microorganisms and plant extracts used as biopreservatives. Microorganisms 11:1896. doi: 10.3390/microorganisms11081896, 37630457 PMC10458850

[ref64] Wang C. Chang T. Yang H. Cui M. (2014). Surface physiological changes induced by lactic acid on pathogens in consideration of pKa and pH. Food Control 46, 525–531. doi: 10.1016/j.foodcont.2014.06.024

[ref65] Wang J. Xu L. Gu L. Lv Y. Li J. Yang Y. . (2023). Cell-free supernatant of *Lactiplantibacillus plantarum* 90: a clean label strategy to improve the shelf life of ground beef gel and its bacteriostatic mechanism. Foods 12:4053. doi: 10.3390/foods12224053, 38002111 PMC10670453

[ref66] Wang G. Zeng H. (2022). Antibacterial effect of cell-free supernatant from *Lactobacillus pentosus* L-36 against *Staphylococcus aureus* from bovine mastitis. Molecules 27:7627. doi: 10.3390/molecules27217627, 36364454 PMC9658419

[ref67] Wemmenhove E. van Valenberg H. J. Zwietering M. H. van Hooijdonk T. C. Wells-Bennik M. H. (2016). Minimal inhibitory concentrations of undissociated lactic, acetic, citric and propionic acid for *Listeria monocytogenes* under conditions relevant to cheese. Food Microbiol. 58, 63–67. doi: 10.1016/j.fm.2016.03.012, 27217360

[ref68] Yildirim S. Röcker B. Pettersen MK. Nilsen‐Nygaar J. Ayhan Z. Rutkaite R. . (2018). Applications of active packaging for food. Compr. Rev. Food Sci. Food Saf. 17, 165–199. doi: 10.1111/1541-4337.12322 33350066

[ref69] Yu W. Guo J. Liu Y. Xue X. Wang X. Wei L. . (2023). Potential impact of combined inhibition by bacteriocins and chemical substances of foodborne pathogenic and spoilage bacteria: a review. Foods 12:3128. doi: 10.3390/foods12163128, 37628127 PMC10453098

[ref70] Zavišić G. Ristić S. Petričević S. Janković D. Petković B. (2024). Microbial contamination of food: probiotics and postbiotics as potential biopreservatives. Foods 13:2487. doi: 10.3390/foods13162487, 39200415 PMC11353716

[ref71] Zhang J. Caiyin Q. Feng W. Zhao X. Qiao B. Zhao G. . (2016). Enhance nisin yield via improving acid-tolerant capability of *Lactococcus lactis* F44. Sci. Rep. 6:27973. doi: 10.1038/srep27973, 27306587 PMC4910042

[ref72] Zhang S. Yao R. Wang Q. Wang W. Zhao S. Wang H. . (2024). Bacteriostatic mechanism of *Lactiplantibacillus plantarum* CS3 cell-free supernatant of on soy sauce spoilage bacteria. Food Biosci. 59:104147. doi: 10.1016/j.fbio.2024.104147

[ref73] Zhao X. Zhao F. Wang J. Zhong N. (2017). Biofilm formation and control strategies of foodborne pathogens: food safety perspectives. RSC Adv. 7, 36670–36683. doi: 10.1039/C7RA02497E

[ref74] Zielińska D. Łepecka A. Ołdak A. Długosz E. Kołożyn-Krajewska D. (2021). Growth and adhesion inhibition of pathogenic bacteria by live and heat-killed food-origin *Lactobacillus* strains or their supernatants. FEMS Microbiol. Lett. 368:fnab024. doi: 10.1093/femsle/fnab024, 33629723

