# Supplementary material for: Bactericidal and antibiofilm activity of lactic acid bacteria-derived cell free extracts against dairy-associated spoilage and pathogenic bacteria
Source: Front Microbiol. 2026 Mar 2;17:1783760. doi: 10.3389/fmicb.2026.1783760 (PMC12989577; doi:10.3389/fmicb.2026.1783760)
Supplement: Supplementary file 1 [file Table_1.docx]

Supplementary Material

**Supplementary Table -** Bacterial normalization values and treatments presenting the lowest Z-score counts.

| *Z - Score* | | | | | | | | | | | | | | | | | | | | | | | | | | |
| --- | --- | --- | --- | --- | --- | --- | --- | --- | --- | --- | --- | --- | --- | --- | --- | --- | --- | --- | --- | --- | --- | --- | --- | --- | --- | --- |
| *590 nm* | | | | | | | | | | | | | | | | | | | | | | | | | | |
| BAC | W8 | W10 | W11 | | W21 | | W22 | W23 | | W25 | W32 | | W42 | | W49 | | ATCC  13675 | | ATCC  19435 | | LC08 | Q4C3 | | SBR4 | |  |
| Σ | 14 | 25 | 31 | | 31 | | 36 | 40 | | 35 | 37 | | 37 | | 31 | | 40 | | 39 | | 41 | 40 | | 31 | |  |
| 600 nm | | | | | | | | | | | | | | | | | | | | | | | | | | |
| Σ | 30 | 31 | 36 | | 41 | | 37 | 37 | | 39 | 36 | | 37 | | 34 | | 41 | | 39 | | 40 | 39 | | 35 | |  |
| *Treatments* | | ***600 nm*** | | | | | | | | | ***590 nm*** | | | | | | | | | | | | | | | |
|  |  | *ICS* | | *ICS N* | | *CFS* | | | *CFS N* | | | *IC* | | *ICS* | | *ICS N* | | *CFS* | | *CFS N* | | | *IC* | |  |  |
| Σ | | *97* | | *94* | | *103* | | | *90* | | | *79* | | *86* | | *83* | | *94* | | *78* | | | 82 | |  |  |

*L. plantarum* Q4C3, *L. lactis* subsp. *lactis* biovar diacetylactis SBR4, *L. lactis* subsp. *lactis* Lc08, *W.* *viridescens* W23, *W. confusa* W8, *W. paramesenteroides* W11, *W. paramesenteroides* W10, *W. cibaria* W42, *W. cibaria* W32, *W. cibaria* W49, *W. cibaria* W21, *W. cibaria* W22, *W. cibaria* W25*, L. lactis* subsp. *lactis* biovar diacetylactis ATCC 13675, *L. lactis* subsp. *lactis* ATCC 19435. CFS: Cell-free supernatants, ICS: inactive cells plus cell-free supernatants, CFS N: neutralized cell free supernatant, ICS N: neutralized inactive cells plus cell-free supernatant and IC: inactive cells.
